# Search for a heavy vector resonance decaying to a ${\mathrm{Z}}_{\mathrm{}}^{\mathrm{}}$  boson and a Higgs boson in proton-proton collisions at $\sqrt{s} = 13\,\text {Te}\text {V}$

**DOI:** 10.1140/epjc/s10052-021-09348-6

**Published:** 2021-08-03

**Authors:** A. M. Sirunyan, A. Tumasyan, W. Adam, T. Bergauer, M. Dragicevic, J. Erö, A. Escalante Del Valle, R. Frühwirth, M. Jeitler, N. Krammer, L. Lechner, D. Liko, I. Mikulec, F. M. Pitters, N. Rad, J. Schieck, R. Schöfbeck, M. Spanring, S. Templ, W. Waltenberger, C.-E. Wulz, M. Zarucki, V. Chekhovsky, A. Litomin, V. Makarenko, J. Suarez Gonzalez, M. R. Darwish, E. A. De Wolf, D. Di Croce, X. Janssen, T. Kello, A. Lelek, M. Pieters, H. Rejeb Sfar, H. Van Haevermaet, P. Van Mechelen, S. Van Putte, N. Van Remortel, F. Blekman, E. S. Bols, S. S. Chhibra, J. D’Hondt, J. De Clercq, D. Lontkovskyi, S. Lowette, I. Marchesini, S. Moortgat, A. Morton, D. Müller, Q. Python, S. Tavernier, W. Van Doninck, P. Van Mulders, D. Beghin, B. Bilin, B. Clerbaux, G. De Lentdecker, B. Dorney, L. Favart, A. Grebenyuk, A. K. Kalsi, I. Makarenko, L. Moureaux, L. Pétré, A. Popov, N. Postiau, E. Starling, L. Thomas, C. Vander Velde, P. Vanlaer, D. Vannerom, L. Wezenbeek, T. Cornelis, D. Dobur, M. Gruchala, I. Khvastunov, M. Niedziela, C. Roskas, K. Skovpen, M. Tytgat, W. Verbeke, B. Vermassen, M. Vit, G. Bruno, F. Bury, C. Caputo, P. David, C. Delaere, M. Delcourt, I. S. Donertas, A. Giammanco, V. Lemaitre, K. Mondal, J. Prisciandaro, A. Taliercio, M. Teklishyn, P. Vischia, S. Wertz, S. Wuyckens, G. A. Alves, C. Hensel, A. Moraes, W. L. Aldá Júnior, E. Belchior Batista Das Chagas, H. Brandao Malbouisson, W. Carvalho, J. Chinellato, E. Coelho, E. M. Da Costa, G. G. Da Silveira, D. De Jesus Damiao, S. Fonseca De Souza, J. Martins, D. Matos Figueiredo, M. Medina Jaime, C. Mora Herrera, L. Mundim, H. Nogima, P. Rebello Teles, L. J. Sanchez Rosas, A. Santoro, S. M. Silva Do Amaral, A. Sznajder, M. Thiel, F. Torres Da Silva De Araujo, A. Vilela Pereira, C. A. Bernardes, L. Calligaris, T. R. Fernandez Perez Tomei, E. M. Gregores, D. S. Lemos, P. G. Mercadante, S. F. Novaes, Sandra S. Padula, A. Aleksandrov, G. Antchev, I. Atanasov, R. Hadjiiska, P. Iaydjiev, M. Misheva, M. Rodozov, M. Shopova, G. Sultanov, A. Dimitrov, T. Ivanov, L. Litov, B. Pavlov, P. Petkov, A. Petrov, T. Cheng, W. Fang, Q. Guo, H. Wang, L. Yuan, M. Ahmad, G. Bauer, Z. Hu, Y. Wang, K. Yi, E. Chapon, G. M. Chen, H. S. Chen, M. Chen, T. Javaid, A. Kapoor, D. Leggat, H. Liao, Z.-A. Liu, R. Sharma, A. Spiezia, J. Tao, J. Thomas-wilsker, J. Wang, H. Zhang, S. Zhang, J. Zhao, A. Agapitos, Y. Ban, C. Chen, Q. Huang, A. Levin, Q. Li, M. Lu, X. Lyu, Y. Mao, S. J. Qian, D. Wang, Q. Wang, J. Xiao, Z. You, X. Gao, M. Xiao, C. Avila, A. Cabrera, C. Florez, J. Fraga, A. Sarkar, M. A. Segura Delgado, J. Jaramillo, J. Mejia Guisao, F. Ramirez, J. D. Ruiz Alvarez, C. A. Salazar González, N. Vanegas Arbelaez, D. Giljanovic, N. Godinovic, D. Lelas, I. Puljak, Z. Antunovic, M. Kovac, T. Sculac, V. Brigljevic, D. Ferencek, D. Majumder, M. Roguljic, A. Starodumov, T. Susa, M. W. Ather, A. Attikis, E. Erodotou, A. Ioannou, G. Kole, M. Kolosova, S. Konstantinou, J. Mousa, C. Nicolaou, F. Ptochos, P. A. Razis, H. Rykaczewski, H. Saka, D. Tsiakkouri, M. Finger, M. Finger, A. Kveton, J. Tomsa, E. Ayala, E. Carrera Jarrin, S. Abu Zeid, Y. Assran, E. Salama, A. Lotfy, M. A. Mahmoud, S. Bhowmik, A. Carvalho Antunes De Oliveira, R. K. Dewanjee, K. Ehataht, M. Kadastik, M. Raidal, C. Veelken, P. Eerola, L. Forthomme, H. Kirschenmann, K. Osterberg, M. Voutilainen, E. Brücken, F. Garcia, J. Havukainen, V. Karimäki, M. S. Kim, R. Kinnunen, T. Lampén, K. Lassila-Perini, S. Lehti, T. Lindén, H. Siikonen, E. Tuominen, J. Tuominiemi, P. Luukka, T. Tuuva, C. Amendola, M. Besancon, F. Couderc, M. Dejardin, D. Denegri, J. L. Faure, F. Ferri, S. Ganjour, A. Givernaud, P. Gras, G. Hamel de Monchenault, P. Jarry, B. Lenzi, E. Locci, J. Malcles, J. Rander, A. Rosowsky, M.Ö. Sahin, A. Savoy-Navarro, M. Titov, G. B. Yu, S. Ahuja, F. Beaudette, M. Bonanomi, A. Buchot Perraguin, P. Busson, C. Charlot, O. Davignon, B. Diab, G. Falmagne, R. Granier de Cassagnac, A. Hakimi, I. Kucher, A. Lobanov, C. Martin Perez, M. Nguyen, C. Ochando, P. Paganini, J. Rembser, R. Salerno, J. B. Sauvan, Y. Sirois, A. Zabi, A. Zghiche, J.-L. Agram, J. Andrea, D. Bloch, G. Bourgatte, J.-M. Brom, E. C. Chabert, C. Collard, J.-C. Fontaine, D. Gelé, U. Goerlach, C. Grimault, A.-C. Le Bihan, P. Van Hove, E. Asilar, S. Beauceron, C. Bernet, G. Boudoul, C. Camen, A. Carle, N. Chanon, D. Contardo, P. Depasse, H. El Mamouni, J. Fay, S. Gascon, M. Gouzevitch, B. Ille, Sa. Jain, I. B. Laktineh, H. Lattaud, A. Lesauvage, M. Lethuillier, L. Mirabito, K. Shchablo, L. Torterotot, G. Touquet, M. Vander Donckt, S. Viret, G. Adamov, Z. Tsamalaidze, L. Feld, K. Klein, M. Lipinski, D. Meuser, A. Pauls, M. Preuten, M. P. Rauch, J. Schulz, M. Teroerde, D. Eliseev, M. Erdmann, P. Fackeldey, B. Fischer, S. Ghosh, T. Hebbeker, K. Hoepfner, H. Keller, L. Mastrolorenzo, M. Merschmeyer, A. Meyer, G. Mocellin, S. Mondal, S. Mukherjee, D. Noll, A. Novak, T. Pook, A. Pozdnyakov, Y. Rath, H. Reithler, J. Roemer, A. Schmidt, S. C. Schuler, A. Sharma, S. Wiedenbeck, S. Zaleski, C. Dziwok, G. Flügge, W. Haj Ahmad, O. Hlushchenko, T. Kress, A. Nowack, C. Pistone, O. Pooth, D. Roy, H. Sert, A. Stahl, T. Ziemons, H. Aarup Petersen, M. Aldaya Martin, P. Asmuss, I. Babounikau, S. Baxter, O. Behnke, A. Bermúdez Martínez, A. A. Bin Anuar, K. Borras, V. Botta, D. Brunner, A. Campbell, A. Cardini, P. Connor, S. Consuegra Rodríguez, V. Danilov, A. De Wit, M. M. Defranchis, L. Didukh, D. Domínguez Damiani, G. Eckerlin, D. Eckstein, T. Eichhorn, L. I. Estevez Banos, E. Gallo, A. Geiser, A. Giraldi, A. Grohsjean, M. Guthoff, A. Harb, A. Jafari, N. Z. Jomhari, H. Jung, A. Kasem, M. Kasemann, H. Kaveh, C. Kleinwort, J. Knolle, D. Krücker, W. Lange, T. Lenz, J. Lidrych, K. Lipka, W. Lohmann, T. Madlener, R. Mankel, I.-A. Melzer-Pellmann, J. Metwally, A. B. Meyer, M. Meyer, M. Missiroli, J. Mnich, A. Mussgiller, V. Myronenko, Y. Otarid, D. Pérez Adán, S. K. Pflitsch, D. Pitzl, A. Raspereza, A. Saggio, A. Saibel, M. Savitskyi, V. Scheurer, C. Schwanenberger, A. Singh, R. E. Sosa Ricardo, N. Tonon, O. Turkot, A. Vagnerini, M. Van De Klundert, R. Walsh, D. Walter, Y. Wen, K. Wichmann, C. Wissing, S. Wuchterl, O. Zenaiev, R. Zlebcik, R. Aggleton, S. Bein, L. Benato, A. Benecke, K. De Leo, T. Dreyer, A. Ebrahimi, M. Eich, F. Feindt, A. Fröhlich, C. Garbers, E. Garutti, P. Gunnellini, J. Haller, A. Hinzmann, A. Karavdina, G. Kasieczka, R. Klanner, R. Kogler, V. Kutzner, J. Lange, T. Lange, A. Malara, C. E. N. Niemeyer, A. Nigamova, K. J. Pena Rodriguez, O. Rieger, P. Schleper, S. Schumann, J. Schwandt, D. Schwarz, J. Sonneveld, H. Stadie, G. Steinbrück, B. Vormwald, I. Zoi, J. Bechtel, T. Berger, E. Butz, R. Caspart, T. Chwalek, W. De Boer, A. Dierlamm, A. Droll, K. El Morabit, N. Faltermann, K. Flöh, M. Giffels, A. Gottmann, F. Hartmann, C. Heidecker, U. Husemann, I. Katkov, P. Keicher, R. Koppenhöfer, S. Maier, M. Metzler, S. Mitra, Th. Müller, M. Musich, G. Quast, K. Rabbertz, J. Rauser, D. Savoiu, D. Schäfer, M. Schnepf, M. Schröder, D. Seith, I. Shvetsov, H. J. Simonis, R. Ulrich, M. Wassmer, M. Weber, R. Wolf, S. Wozniewski, G. Anagnostou, P. Asenov, G. Daskalakis, T. Geralis, A. Kyriakis, D. Loukas, G. Paspalaki, A. Stakia, M. Diamantopoulou, D. Karasavvas, G. Karathanasis, P. Kontaxakis, C. K. Koraka, A. Manousakis-katsikakis, A. Panagiotou, I. Papavergou, N. Saoulidou, K. Theofilatos, E. Tziaferi, K. Vellidis, E. Vourliotis, G. Bakas, K. Kousouris, I. Papakrivopoulos, G. Tsipolitis, A. Zacharopoulou, I. Evangelou, C. Foudas, P. Gianneios, P. Katsoulis, P. Kokkas, K. Manitara, N. Manthos, I. Papadopoulos, J. Strologas, M. Bartók, M. Csanad, M. M. A. Gadallah, S. Lökös, P. Major, K. Mandal, A. Mehta, G. Pasztor, O. Surányi, G. I. Veres, G. Bencze, C. Hajdu, D. Horvath, F. Sikler, V. Veszpremi, G. Vesztergombi, S. Czellar, J. Karancsi, J. Molnar, Z. Szillasi, D. Teyssier, P. Raics, Z. L. Trocsanyi, B. Ujvari, T. Csorgo, F. Nemes, T. Novak, S. Choudhury, J. R. Komaragiri, D. Kumar, L. Panwar, P. C. Tiwari, S. Bahinipati, D. Dash, C. Kar, P. Mal, T. Mishra, V. K. Muraleedharan Nair Bindhu, A. Nayak, D. K. Sahoo, N. Sur, S. K. Swain, S. Bansal, S. B. Beri, V. Bhatnagar, G. Chaudhary, S. Chauhan, N. Dhingra, R. Gupta, A. Kaur, S. Kaur, P. Kumari, M. Meena, K. Sandeep, S. Sharma, J. B. Singh, A. K. Virdi, A. Ahmed, A. Bhardwaj, B. C. Choudhary, R. B. Garg, M. Gola, S. Keshri, A. Kumar, M. Naimuddin, P. Priyanka, K. Ranjan, A. Shah, M. Bharti, R. Bhattacharya, S. Bhattacharya, D. Bhowmik, S. Dutta, S. Ghosh, B. Gomber, M. Maity, S. Nandan, P. Palit, P. K. Rout, G. Saha, B. Sahu, S. Sarkar, M. Sharan, B. Singh, S. Thakur, P. K. Behera, S. C. Behera, P. Kalbhor, A. Muhammad, R. Pradhan, P. R. Pujahari, A. Sharma, A. K. Sikdar, D. Dutta, V. Kumar, K. Naskar, P. K. Netrakanti, L. M. Pant, P. Shukla, T. Aziz, M. A. Bhat, S. Dugad, R. Kumar Verma, G. B. Mohanty, U. Sarkar, S. Banerjee, S. Bhattacharya, S. Chatterjee, R. Chudasama, M. Guchait, S. Karmakar, S. Kumar, G. Majumder, K. Mazumdar, S. Mukherjee, D. Roy, S. Dube, B. Kansal, S. Pandey, A. Rane, A. Rastogi, S. Sharma, H. Bakhshiansohi, M. Zeinali, S. Chenarani, S. M. Etesami, M. Khakzad, M. Mohammadi Najafabadi, M. Felcini, M. Grunewald, M. Abbrescia, R. Aly, C. Aruta, A. Colaleo, D. Creanza, N. De Filippis, M. De Palma, A. Di Florio, A. Di Pilato, W. Elmetenawee, L. Fiore, A. Gelmi, M. Gul, G. Iaselli, M. Ince, S. Lezki, G. Maggi, M. Maggi, I. Margjeka, V. Mastrapasqua, J. A. Merlin, S. My, S. Nuzzo, A. Pompili, G. Pugliese, A. Ranieri, G. Selvaggi, L. Silvestris, F. M. Simone, R. Venditti, P. Verwilligen, G. Abbiendi, C. Battilana, D. Bonacorsi, L. Borgonovi, S. Braibant-Giacomelli, R. Campanini, P. Capiluppi, A. Castro, F. R. Cavallo, C. Ciocca, M. Cuffiani, G. M. Dallavalle, T. Diotalevi, F. Fabbri, A. Fanfani, E. Fontanesi, P. Giacomelli, L. Giommi, C. Grandi, L. Guiducci, F. Iemmi, S. Lo Meo, S. Marcellini, G. Masetti, F. L. Navarria, A. Perrotta, F. Primavera, A. M. Rossi, T. Rovelli, G. P. Siroli, N. Tosi, S. Albergo, S. Costa, A. Di Mattia, R. Potenza, A. Tricomi, C. Tuve, G. Barbagli, A. Cassese, R. Ceccarelli, V. Ciulli, C. Civinini, R. D’Alessandro, F. Fiori, E. Focardi, G. Latino, P. Lenzi, M. Lizzo, M. Meschini, S. Paoletti, R. Seidita, G. Sguazzoni, L. Viliani, L. Benussi, S. Bianco, D. Piccolo, M. Bozzo, F. Ferro, R. Mulargia, E. Robutti, S. Tosi, A. Benaglia, A. Beschi, F. Brivio, F. Cetorelli, V. Ciriolo, F. De Guio, M. E. Dinardo, P. Dini, S. Gennai, A. Ghezzi, P. Govoni, L. Guzzi, M. Malberti, S. Malvezzi, A. Massironi, D. Menasce, F. Monti, L. Moroni, M. Paganoni, D. Pedrini, S. Ragazzi, T. Tabarelli de Fatis, D. Valsecchi, D. Zuolo, S. Buontempo, N. Cavallo, A. De Iorio, F. Fabozzi, F. Fienga, A. O. M. Iorio, L. Lista, S. Meola, P. Paolucci, B. Rossi, C. Sciacca, E. Voevodina, P. Azzi, N. Bacchetta, D. Bisello, P. Bortignon, A. Bragagnolo, R. Carlin, P. Checchia, P. De Castro Manzano, T. Dorigo, F. Gasparini, U. Gasparini, S. Y. Hoh, L. Layer, M. Margoni, A. T. Meneguzzo, M. Presilla, P. Ronchese, R. Rossin, F. Simonetto, G. Strong, M. Tosi, H. YARAR, M. Zanetti, P. Zotto, A. Zucchetta, G. Zumerle, C. Aime‘, A. Braghieri, S. Calzaferri, D. Fiorina, P. Montagna, S. P. Ratti, V. Re, M. Ressegotti, C. Riccardi, P. Salvini, I. Vai, P. Vitulo, M. Biasini, G. M. Bilei, D. Ciangottini, L. Fanò, P. Lariccia, G. Mantovani, V. Mariani, M. Menichelli, F. Moscatelli, A. Piccinelli, A. Rossi, A. Santocchia, D. Spiga, T. Tedeschi, K. Androsov, P. Azzurri, G. Bagliesi, V. Bertacchi, L. Bianchini, T. Boccali, R. Castaldi, M. A. Ciocci, R. Dell’Orso, M. R. Di Domenico, S. Donato, L. Giannini, A. Giassi, M. T. Grippo, F. Ligabue, E. Manca, G. Mandorli, A. Messineo, F. Palla, G. Ramirez-Sanchez, A. Rizzi, G. Rolandi, S. Roy Chowdhury, A. Scribano, N. Shafiei, P. Spagnolo, R. Tenchini, G. Tonelli, N. Turini, A. Venturi, P. G. Verdini, F. Cavallari, M. Cipriani, D. Del Re, E. Di Marco, M. Diemoz, E. Longo, P. Meridiani, G. Organtini, F. Pandolfi, R. Paramatti, C. Quaranta, S. Rahatlou, C. Rovelli, F. Santanastasio, L. Soffi, R. Tramontano, N. Amapane, R. Arcidiacono, S. Argiro, M. Arneodo, N. Bartosik, R. Bellan, A. Bellora, J. Berenguer Antequera, C. Biino, A. Cappati, N. Cartiglia, S. Cometti, M. Costa, R. Covarelli, N. Demaria, B. Kiani, F. Legger, C. Mariotti, S. Maselli, E. Migliore, V. Monaco, E. Monteil, M. Monteno, M. M. Obertino, G. Ortona, L. Pacher, N. Pastrone, M. Pelliccioni, G. L. Pinna Angioni, M. Ruspa, R. Salvatico, F. Siviero, V. Sola, A. Solano, D. Soldi, A. Staiano, M. Tornago, D. Trocino, S. Belforte, V. Candelise, M. Casarsa, F. Cossutti, A. Da Rold, G. Della Ricca, F. Vazzoler, S. Dogra, C. Huh, B. Kim, D. H. Kim, G. N. Kim, J. Lee, S. W. Lee, C. S. Moon, Y. D. Oh, S. I. Pak, B. C. Radburn-Smith, S. Sekmen, Y. C. Yang, H. Kim, D. H. Moon, B. Francois, T. J. Kim, J. Park, S. Cho, S. Choi, Y. Go, S. Ha, B. Hong, K. Lee, K. S. Lee, J. Lim, J. Park, S. K. Park, J. Yoo, J. Goh, A. Gurtu, H. S. Kim, Y. Kim, J. Almond, J. H. Bhyun, J. Choi, S. Jeon, J. Kim, J. S. Kim, S. Ko, H. Kwon, H. Lee, K. Lee, S. Lee, K. Nam, B. H. Oh, M. Oh, S. B. Oh, H. Seo, U. K. Yang, I. Yoon, D. Jeon, J. H. Kim, B. Ko, J. S. H. Lee, I. C. Park, Y. Roh, D. Song, I. J. Watson, H. D. Yoo, Y. Choi, C. Hwang, Y. Jeong, H. Lee, Y. Lee, I. Yu, Y. Maghrbi, V. Veckalns, A. Juodagalvis, A. Rinkevicius, G. Tamulaitis, A. Vaitkevicius, W. A. T. Wan Abdullah, M. N. Yusli, Z. Zolkapli, J. F. Benitez, A. Castaneda Hernandez, J. A. Murillo Quijada, L. Valencia Palomo, G. Ayala, H. Castilla-Valdez, E. De La Cruz-Burelo, I. Heredia-De La Cruz, R. Lopez-Fernandez, C. A. Mondragon Herrera, D. A. Perez Navarro, A. Sanchez-Hernandez, S. Carrillo Moreno, C. Oropeza Barrera, M. Ramirez-Garcia, F. Vazquez Valencia, J. Eysermans, I. Pedraza, H. A. Salazar Ibarguen, C. Uribe Estrada, A. Morelos Pineda, J. Mijuskovic, N. Raicevic, D. Krofcheck, S. Bheesette, P. H. Butler, A. Ahmad, M. I. Asghar, A. Awais, M. I. M. Awan, H. R. Hoorani, W. A. Khan, M. A. Shah, M. Shoaib, M. Waqas, V. Avati, L. Grzanka, M. Malawski, H. Bialkowska, M. Bluj, B. Boimska, T. Frueboes, M. Górski, M. Kazana, M. Szleper, P. Traczyk, P. Zalewski, K. Bunkowski, K. Doroba, A. Kalinowski, M. Konecki, J. Krolikowski, M. Walczak, M. Araujo, P. Bargassa, D. Bastos, A. Boletti, P. Faccioli, M. Gallinaro, J. Hollar, N. Leonardo, T. Niknejad, J. Seixas, K. Shchelina, O. Toldaiev, J. Varela, S. Afanasiev, P. Bunin, M. Gavrilenko, I. Golutvin, A. Kamenev, V. Karjavine, I. Kashunin, V. Korenkov, A. Lanev, A. Malakhov, V. Matveev, V. V. Mitsyn, V. Palichik, V. Perelygin, M. Savina, V. Shalaev, S. Shmatov, S. Shulha, V. Smirnov, O. Teryaev, V. Trofimov, A. Zarubin, G. Gavrilov, V. Golovtcov, Y. Ivanov, V. Kim, E. Kuznetsova, V. Murzin, V. Oreshkin, I. Smirnov, D. Sosnov, V. Sulimov, L. Uvarov, S. Volkov, A. Vorobyev, Yu. Andreev, A. Dermenev, S. Gninenko, N. Golubev, A. Karneyeu, M. Kirsanov, N. Krasnikov, A. Pashenkov, G. Pivovarov, D. Tlisov, A. Toropin, V. Epshteyn, V. Gavrilov, N. Lychkovskaya, A. Nikitenko, V. Popov, G. Safronov, A. Spiridonov, A. Stepennov, M. Toms, E. Vlasov, A. Zhokin, T. Aushev, O. Bychkova, D. Philippov, E. Popova, V. Rusinov, E. Zhemchugov, V. Andreev, M. Azarkin, I. Dremin, M. Kirakosyan, A. Terkulov, A. Belyaev, E. Boos, V. Bunichev, M. Dubinin, L. Dudko, A. Ershov, V. Klyukhin, O. Kodolova, I. Lokhtin, S. Obraztsov, M. Perfilov, S. Petrushanko, V. Savrin, V. Blinov, T. Dimova, L. Kardapoltsev, I. Ovtin, Y. Skovpen, I. Azhgirey, I. Bayshev, V. Kachanov, A. Kalinin, D. Konstantinov, V. Petrov, R. Ryutin, A. Sobol, S. Troshin, N. Tyurin, A. Uzunian, A. Volkov, A. Babaev, A. Iuzhakov, V. Okhotnikov, L. Sukhikh, V. Borchsh, V. Ivanchenko, E. Tcherniaev, P. Adzic, P. Cirkovic, M. Dordevic, P. Milenovic, J. Milosevic, M. Aguilar-Benitez, J. Alcaraz Maestre, A. Álvarez Fernández, I. Bachiller, M. Barrio Luna, Cristina F. Bedoya, C. A. Carrillo Montoya, M. Cepeda, M. Cerrada, N. Colino, B. De La Cruz, A. Delgado Peris, J. P. Fernández Ramos, J. Flix, M. C. Fouz, O. Gonzalez Lopez, S. Goy Lopez, J. M. Hernandez, M. I. Josa, J. León Holgado, D. Moran, Á. Navarro Tobar, A. Pérez-Calero Yzquierdo, J. Puerta Pelayo, I. Redondo, L. Romero, S. Sánchez Navas, M. S. Soares, A. Triossi, L. Urda Gómez, C. Willmott, C. Albajar, J. F. de Trocóniz, R. Reyes-Almanza, B. Alvarez Gonzalez, J. Cuevas, C. Erice, J. Fernandez Menendez, S. Folgueras, I. Gonzalez Caballero, E. Palencia Cortezon, C. Ramón Álvarez, J. Ripoll Sau, V. Rodríguez Bouza, S. Sanchez Cruz, A. Trapote, J. A. Brochero Cifuentes, I. J. Cabrillo, A. Calderon, B. Chazin Quero, J. Duarte Campderros, M. Fernandez, P. J. Fernández Manteca, A. García Alonso, G. Gomez, C. Martinez Rivero, P. Martinez Ruiz del Arbol, F. Matorras, J. Piedra Gomez, C. Prieels, F. Ricci-Tam, T. Rodrigo, A. Ruiz-Jimeno, L. Scodellaro, I. Vila, J. M. Vizan Garcia, MK Jayananda, B. Kailasapathy, D. U. J. Sonnadara, DDC Wickramarathna, W. G. D. Dharmaratna, K. Liyanage, N. Perera, N. Wickramage, T. K. Aarrestad, D. Abbaneo, E. Auffray, G. Auzinger, J. Baechler, P. Baillon, A. H. Ball, D. Barney, J. Bendavid, N. Beni, M. Bianco, A. Bocci, E. Bossini, E. Brondolin, T. Camporesi, M. Capeans Garrido, G. Cerminara, L. Cristella, D. d’Enterria, A. Dabrowski, N. Daci, V. Daponte, A. David, A. De Roeck, M. Deile, R. Di Maria, M. Dobson, M. Dünser, N. Dupont, A. Elliott-Peisert, N. Emriskova, F. Fallavollita, D. Fasanella, S. Fiorendi, A. Florent, G. Franzoni, J. Fulcher, W. Funk, S. Giani, D. Gigi, K. Gill, F. Glege, L. Gouskos, M. Guilbaud, D. Gulhan, M. Haranko, J. Hegeman, Y. Iiyama, V. Innocente, T. James, P. Janot, J. Kaspar, J. Kieseler, M. Komm, N. Kratochwil, C. Lange, S. Laurila, P. Lecoq, K. Long, C. Lourenço, L. Malgeri, S. Mallios, M. Mannelli, F. Meijers, S. Mersi, E. Meschi, F. Moortgat, M. Mulders, S. Orfanelli, L. Orsini, F. Pantaleo, L. Pape, E. Perez, M. Peruzzi, A. Petrilli, G. Petrucciani, A. Pfeiffer, M. Pierini, T. Quast, D. Rabady, A. Racz, M. Rieger, M. Rovere, H. Sakulin, J. Salfeld-Nebgen, S. Scarfi, C. Schäfer, C. Schwick, M. Selvaggi, A. Sharma, P. Silva, W. Snoeys, P. Sphicas, S. Summers, V. R. Tavolaro, D. Treille, A. Tsirou, G. P. Van Onsem, A. Vartak, M. Verzetti, K. A. Wozniak, W. D. Zeuner, L. Caminada, W. Erdmann, R. Horisberger, Q. Ingram, H. C. Kaestli, D. Kotlinski, U. Langenegger, T. Rohe, M. Backhaus, P. Berger, A. Calandri, N. Chernyavskaya, A. De Cosa, G. Dissertori, M. Dittmar, M. Donegà, C. Dorfer, T. Gadek, T. A. Gómez Espinosa, C. Grab, D. Hits, W. Lustermann, A.-M. Lyon, R. A. Manzoni, M. T. Meinhard, F. Micheli, F. Nessi-Tedaldi, J. Niedziela, F. Pauss, V. Perovic, G. Perrin, S. Pigazzini, M. G. Ratti, M. Reichmann, C. Reissel, T. Reitenspiess, B. Ristic, D. Ruini, D. A. Sanz Becerra, M. Schönenberger, V. Stampf, J. Steggemann, M. L. Vesterbacka Olsson, R. Wallny, D. H. Zhu, C. Amsler, P. Bärtschi, C. Botta, D. Brzhechko, M. F. Canelli, R. Del Burgo, J. K. Heikkilä, M. Huwiler, A. Jofrehei, B. Kilminster, S. Leontsinis, A. Macchiolo, P. Meiring, V. M. Mikuni, U. Molinatti, I. Neutelings, G. Rauco, A. Reimers, P. Robmann, K. Schweiger, Y. Takahashi, C. Adloff, C. M. Kuo, W. Lin, A. Roy, T. Sarkar, S. S. Yu, L. Ceard, P. Chang, Y. Chao, K. F. Chen, P. H. Chen, W.-S. Hou, Y. y. Li, R.-S. Lu, E. Paganis, A. Psallidas, A. Steen, E. Yazgan, B. Asavapibhop, C. Asawatangtrakuldee, N. Srimanobhas, M. N. Bakirci, F. Boran, S. Damarseckin, Z. S. Demiroglu, F. Dolek, C. Dozen, I. Dumanoglu, E. Eskut, Y. Guler, E. Gurpinar Guler, I. Hos, C. Isik, E. E. Kangal, O. Kara, A. Kayis Topaksu, U. Kiminsu, G. Onengut, A. Polatoz, A. E. Simsek, B. Tali, U. G. Tok, H. Topakli, S. Turkcapar, I. S. Zorbakir, C. Zorbilmez, B. Isildak, G. Karapinar, K. Ocalan, M. Yalvac, B. Akgun, I. O. Atakisi, E. Gülmez, M. Kaya, O. Kaya, Ö. Özçelik, S. Tekten, E. A. Yetkin, A. Cakir, K. Cankocak, Y. Komurcu, S. Sen, F. Aydogmus Sen, S. Cerci, B. Kaynak, S. Ozkorucuklu, D. Sunar Cerci, B. Grynyov, L. Levchuk, E. Bhal, S. Bologna, J. J. Brooke, E. Clement, D. Cussans, H. Flacher, J. Goldstein, G. P. Heath, H. F. Heath, L. Kreczko, B. Krikler, S. Paramesvaran, T. Sakuma, S. Seif El Nasr-Storey, V. J. Smith, N. Stylianou, J. Taylor, A. Titterton, K. W. Bell, A. Belyaev, C. Brew, R. M. Brown, D. J. A. Cockerill, K. V. Ellis, K. Harder, S. Harper, J. Linacre, K. Manolopoulos, D. M. Newbold, E. Olaiya, D. Petyt, T. Reis, T. Schuh, C. H. Shepherd-Themistocleous, A. Thea, I. R. Tomalin, T. Williams, R. Bainbridge, P. Bloch, S. Bonomally, J. Borg, S. Breeze, O. Buchmuller, A. Bundock, V. Cepaitis, G. S. Chahal, D. Colling, P. Dauncey, G. Davies, M. Della Negra, G. Fedi, G. Hall, G. Iles, J. Langford, L. Lyons, A.-M. Magnan, S. Malik, A. Martelli, V. Milosevic, J. Nash, V. Palladino, M. Pesaresi, D. M. Raymond, A. Richards, A. Rose, E. Scott, C. Seez, A. Shtipliyski, M. Stoye, A. Tapper, K. Uchida, T. Virdee, N. Wardle, S. N. Webb, D. Winterbottom, A. G. Zecchinelli, J. E. Cole, P. R. Hobson, A. Khan, P. Kyberd, C. K. Mackay, I. D. Reid, L. Teodorescu, S. Zahid, S. Abdullin, A. Brinkerhoff, K. Call, B. Caraway, J. Dittmann, K. Hatakeyama, A. R. Kanuganti, C. Madrid, B. McMaster, N. Pastika, S. Sawant, C. Smith, J. Wilson, R. Bartek, A. Dominguez, R. Uniyal, A. M. Vargas Hernandez, A. Buccilli, O. Charaf, S. I. Cooper, S. V. Gleyzer, C. Henderson, C. U. Perez, P. Rumerio, C. West, A. Akpinar, A. Albert, D. Arcaro, C. Cosby, Z. Demiragli, D. Gastler, J. Rohlf, K. Salyer, D. Sperka, D. Spitzbart, I. Suarez, S. Yuan, D. Zou, G. Benelli, B. Burkle, X. Coubez, D. Cutts, Y. t. Duh, M. Hadley, U. Heintz, J. M. Hogan, K. H. M. Kwok, E. Laird, G. Landsberg, K. T. Lau, J. Lee, M. Narain, S. Sagir, R. Syarif, E. Usai, W. Y. Wong, D. Yu, W. Zhang, R. Band, C. Brainerd, R. Breedon, M. Calderon De La Barca Sanchez, M. Chertok, J. Conway, R. Conway, P. T. Cox, R. Erbacher, C. Flores, G. Funk, F. Jensen, W. Ko, O. Kukral, R. Lander, M. Mulhearn, D. Pellett, J. Pilot, M. Shi, D. Taylor, K. Tos, M. Tripathi, Y. Yao, F. Zhang, M. Bachtis, R. Cousins, A. Dasgupta, D. Hamilton, J. Hauser, M. Ignatenko, M. A. Iqbal, T. Lam, N. Mccoll, W. A. Nash, S. Regnard, D. Saltzberg, C. Schnaible, B. Stone, V. Valuev, K. Burt, Y. Chen, R. Clare, J. W. Gary, G. Hanson, G. Karapostoli, O. R. Long, N. Manganelli, M. Olmedo Negrete, M. I. Paneva, W. Si, S. Wimpenny, Y. Zhang, J. G. Branson, P. Chang, S. Cittolin, S. Cooperstein, N. Deelen, J. Duarte, R. Gerosa, D. Gilbert, V. Krutelyov, J. Letts, M. Masciovecchio, S. May, S. Padhi, M. Pieri, V. Sharma, M. Tadel, F. Würthwein, A. Yagil, N. Amin, C. Campagnari, M. Citron, A. Dorsett, V. Dutta, J. Incandela, B. Marsh, H. Mei, A. Ovcharova, H. Qu, M. Quinnan, J. Richman, U. Sarica, D. Stuart, S. Wang, A. Bornheim, O. Cerri, I. Dutta, J. M. Lawhorn, N. Lu, J. Mao, H. B. Newman, J. Ngadiuba, T. Q. Nguyen, J. Pata, M. Spiropulu, J. R. Vlimant, C. Wang, S. Xie, Z. Zhang, R. Y. Zhu, J. Alison, M. B. Andrews, T. Ferguson, T. Mudholkar, M. Paulini, M. Sun, I. Vorobiev, J. P. Cumalat, W. T. Ford, E. MacDonald, T. Mulholland, R. Patel, A. Perloff, K. Stenson, K. A. Ulmer, S. R. Wagner, J. Alexander, Y. Cheng, J. Chu, D. J. Cranshaw, A. Datta, A. Frankenthal, K. Mcdermott, J. Monroy, J. R. Patterson, D. Quach, A. Ryd, W. Sun, S. M. Tan, Z. Tao, J. Thom, P. Wittich, M. Zientek, M. Albrow, M. Alyari, G. Apollinari, A. Apresyan, A. Apyan, S. Banerjee, L. A. T. Bauerdick, A. Beretvas, D. Berry, J. Berryhill, P. C. Bhat, K. Burkett, J. N. Butler, A. Canepa, G. B. Cerati, H. W. K. Cheung, F. Chlebana, M. Cremonesi, V. D. Elvira, J. Freeman, Z. Gecse, E. Gottschalk, L. Gray, D. Green, S. Grünendahl, O. Gutsche, R. M. Harris, S. Hasegawa, R. Heller, T. C. Herwig, J. Hirschauer, B. Jayatilaka, S. Jindariani, M. Johnson, U. Joshi, P. Klabbers, T. Klijnsma, B. Klima, M. J. Kortelainen, S. Lammel, D. Lincoln, R. Lipton, M. Liu, T. Liu, J. Lykken, K. Maeshima, D. Mason, P. McBride, P. Merkel, S. Mrenna, S. Nahn, V. O’Dell, V. Papadimitriou, K. Pedro, C. Pena, O. Prokofyev, F. Ravera, A. Reinsvold Hall, L. Ristori, B. Schneider, E. Sexton-Kennedy, N. Smith, A. Soha, W. J. Spalding, L. Spiegel, S. Stoynev, J. Strait, L. Taylor, S. Tkaczyk, N. V. Tran, L. Uplegger, E. W. Vaandering, H. A. Weber, A. Woodard, D. Acosta, P. Avery, D. Bourilkov, L. Cadamuro, V. Cherepanov, F. Errico, R. D. Field, D. Guerrero, B. M. Joshi, M. Kim, J. Konigsberg, A. Korytov, K. H. Lo, K. Matchev, N. Menendez, G. Mitselmakher, D. Rosenzweig, K. Shi, J. Sturdy, J. Wang, S. Wang, X. Zuo, T. Adams, A. Askew, D. Diaz, R. Habibullah, S. Hagopian, V. Hagopian, K. F. Johnson, R. Khurana, T. Kolberg, G. Martinez, H. Prosper, C. Schiber, R. Yohay, J. Zhang, M. M. Baarmand, S. Butalla, T. Elkafrawy, M. Hohlmann, D. Noonan, M. Rahmani, M. Saunders, F. Yumiceva, M. R. Adams, L. Apanasevich, H. Becerril Gonzalez, R. Cavanaugh, X. Chen, S. Dittmer, O. Evdokimov, C. E. Gerber, D. A. Hangal, D. J. Hofman, C. Mills, G. Oh, T. Roy, M. B. Tonjes, N. Varelas, J. Viinikainen, X. Wang, Z. Wu, Z. Ye, M. Alhusseini, K. Dilsiz, S. Durgut, R. P. Gandrajula, M. Haytmyradov, V. Khristenko, O. K. Köseyan, J.-P. Merlo, A. Mestvirishvili, A. Moeller, J. Nachtman, H. Ogul, Y. Onel, F. Ozok, A. Penzo, C. Snyder, E. Tiras, J. Wetzel, O. Amram, B. Blumenfeld, L. Corcodilos, M. Eminizer, A. V. Gritsan, S. Kyriacou, P. Maksimovic, C. Mantilla, J. Roskes, M. Swartz, T. Á. Vámi, C. Baldenegro Barrera, P. Baringer, A. Bean, A. Bylinkin, T. Isidori, S. Khalil, J. King, G. Krintiras, A. Kropivnitskaya, C. Lindsey, N. Minafra, M. Murray, C. Rogan, C. Royon, S. Sanders, E. Schmitz, J. D. Tapia Takaki, Q. Wang, J. Williams, G. Wilson, S. Duric, A. Ivanov, K. Kaadze, D. Kim, Y. Maravin, T. Mitchell, A. Modak, A. Mohammadi, F. Rebassoo, D. Wright, E. Adams, A. Baden, O. Baron, A. Belloni, S. C. Eno, Y. Feng, N. J. Hadley, S. Jabeen, G. Y. Jeng, R. G. Kellogg, T. Koeth, A. C. Mignerey, S. Nabili, M. Seidel, A. Skuja, S. C. Tonwar, L. Wang, K. Wong, D. Abercrombie, B. Allen, R. Bi, S. Brandt, W. Busza, I. A. Cali, Y. Chen, M. D’Alfonso, G. Gomez Ceballos, M. Goncharov, P. Harris, D. Hsu, M. Hu, M. Klute, D. Kovalskyi, J. Krupa, Y.-J. Lee, P. D. Luckey, B. Maier, A. C. Marini, C. Mcginn, C. Mironov, S. Narayanan, X. Niu, C. Paus, D. Rankin, C. Roland, G. Roland, Z. Shi, G. S. F. Stephans, K. Sumorok, K. Tatar, D. Velicanu, J. Wang, T. W. Wang, Z. Wang, B. Wyslouch, R. M. Chatterjee, A. Evans, P. Hansen, J. Hiltbrand, Sh. Jain, M. Krohn, Y. Kubota, Z. Lesko, J. Mans, M. Revering, R. Rusack, R. Saradhy, N. Schroeder, N. Strobbe, M. A. Wadud, J. G. Acosta, S. Oliveros, K. Bloom, S. Chauhan, D. R. Claes, C. Fangmeier, L. Finco, F. Golf, J. R. González Fernández, C. Joo, I. Kravchenko, J. E. Siado, G. R. Snow, W. Tabb, F. Yan, G. Agarwal, H. Bandyopadhyay, C. Harrington, L. Hay, I. Iashvili, A. Kharchilava, C. McLean, D. Nguyen, J. Pekkanen, S. Rappoccio, B. Roozbahani, G. Alverson, E. Barberis, C. Freer, Y. Haddad, A. Hortiangtham, J. Li, G. Madigan, B. Marzocchi, D. M. Morse, V. Nguyen, T. Orimoto, A. Parker, L. Skinnari, A. Tishelman-Charny, T. Wamorkar, B. Wang, A. Wisecarver, D. Wood, S. Bhattacharya, J. Bueghly, Z. Chen, A. Gilbert, T. Gunter, K. A. Hahn, N. Odell, M. H. Schmitt, K. Sung, M. Velasco, R. Bucci, N. Dev, R. Goldouzian, M. Hildreth, K. Hurtado Anampa, C. Jessop, D. J. Karmgard, K. Lannon, N. Loukas, N. Marinelli, I. Mcalister, F. Meng, K. Mohrman, Y. Musienko, R. Ruchti, P. Siddireddy, S. Taroni, M. Wayne, A. Wightman, M. Wolf, L. Zygala, J. Alimena, B. Bylsma, B. Cardwell, L. S. Durkin, B. Francis, C. Hill, A. Lefeld, B. L. Winer, B. R. Yates, B. Bonham, P. Das, G. Dezoort, P. Elmer, B. Greenberg, N. Haubrich, S. Higginbotham, A. Kalogeropoulos, G. Kopp, S. Kwan, D. Lange, M. T. Lucchini, J. Luo, D. Marlow, K. Mei, I. Ojalvo, J. Olsen, C. Palmer, P. Piroué, D. Stickland, C. Tully, S. Malik, S. Norberg, V. E. Barnes, R. Chawla, S. Das, L. Gutay, M. Jones, A. W. Jung, G. Negro, N. Neumeister, C. C. Peng, S. Piperov, A. Purohit, H. Qiu, J. F. Schulte, M. Stojanovic, N. Trevisani, F. Wang, A. Wildridge, R. Xiao, W. Xie, J. Dolen, N. Parashar, A. Baty, S. Dildick, K. M. Ecklund, S. Freed, F. J. M. Geurts, M. Kilpatrick, A. Kumar, W. Li, B. P. Padley, R. Redjimi, J. Roberts, J. Rorie, W. Shi, A. G. Stahl Leiton, A. Bodek, P. de Barbaro, R. Demina, J. L. Dulemba, C. Fallon, T. Ferbel, M. Galanti, A. Garcia-Bellido, O. Hindrichs, A. Khukhunaishvili, E. Ranken, R. Taus, B. Chiarito, J. P. Chou, A. Gandrakota, Y. Gershtein, E. Halkiadakis, A. Hart, M. Heindl, E. Hughes, S. Kaplan, O. Karacheban, I. Laflotte, A. Lath, R. Montalvo, K. Nash, M. Osherson, S. Salur, S. Schnetzer, S. Somalwar, R. Stone, S. A. Thayil, S. Thomas, H. Wang, H. Acharya, A. G. Delannoy, S. Spanier, O. Bouhali, M. Dalchenko, A. Delgado, R. Eusebi, J. Gilmore, T. Huang, T. Kamon, H. Kim, S. Luo, S. Malhotra, R. Mueller, D. Overton, L. Perniè, D. Rathjens, A. Safonov, N. Akchurin, J. Damgov, V. Hegde, S. Kunori, K. Lamichhane, S. W. Lee, T. Mengke, S. Muthumuni, T. Peltola, S. Undleeb, I. Volobouev, Z. Wang, A. Whitbeck, E. Appelt, S. Greene, A. Gurrola, R. Janjam, W. Johns, C. Maguire, A. Melo, H. Ni, K. Padeken, F. Romeo, P. Sheldon, S. Tuo, J. Velkovska, M. W. Arenton, B. Cox, G. Cummings, J. Hakala, R. Hirosky, M. Joyce, A. Ledovskoy, A. Li, C. Neu, B. Tannenwald, Y. Wang, E. Wolfe, F. Xia, P. E. Karchin, N. Poudyal, P. Thapa, K. Black, T. Bose, J. Buchanan, C. Caillol, S. Dasu, I. De Bruyn, P. Everaerts, C. Galloni, H. He, M. Herndon, A. Hervé, U. Hussain, A. Lanaro, A. Loeliger, R. Loveless, J. Madhusudanan Sreekala, A. Mallampalli, D. Pinna, A. Savin, V. Shang, V. Sharma, W. H. Smith, D. Teague, S. Trembath-reichert, W. Vetens, CMS Collaboration

**Affiliations:** 1 Yerevan Physics Institute, Yerevan, Armenia; 2 Institut für Hochenergiephysik, Vienna, Austria; 3 Institute for Nuclear Problems, Minsk, Belarus; 4 Universiteit Antwerpen, Antwerp, Belgium; 5 Vrije Universiteit Brussel, Brussels, Belgium; 6 Université Libre de Bruxelles, Brussels, Belgium; 7 Ghent University, Ghent, Belgium; 8 Université Catholique de Louvain, Louvain-la-Neuve, Belgium; 9 Centro Brasileiro de Pesquisas Fisicas, Rio de Janeiro, Brazil; 10 Universidade do Estado do Rio de Janeiro, Rio de Janeiro, Brazil; 11 Universidade Estadual Paulista, Universidade Federal do ABC, São Paulo, Brazil; 12 Institute for Nuclear Research and Nuclear Energy, Bulgarian Academy of Sciences, Sofia, Bulgaria; 13 University of Sofia, Sofia, Bulgaria; 14 Beihang University, Beijing, China; 15 Department of Physics, Tsinghua University, Beijing, China; 16 Institute of High Energy Physics, Beijing, China; 17 State Key Laboratory of Nuclear Physics and Technology, Peking University, Beijing, China; 18 Sun Yat-Sen University, Guangzhou, China; 19 Institute of Modern Physics and Key Laboratory of Nuclear Physics and Ion-beam Application (MOE)-Fudan University, Shanghai, China; 20 Zhejiang University, Hangzhou, China; 21 Universidad de Los Andes, Bogotá, Colombia; 22 Universidad de Antioquia, Medellín, Colombia; 23 Faculty of Electrical Engineering, Mechanical Engineering and Naval Architecture, University of Split, Split, Croatia; 24 University of Split, Faculty of Science, Split, Croatia; 25 Institute Rudjer Boskovic, Zagreb, Croatia; 26 University of Cyprus, Nicosia, Cyprus; 27 Charles University, Prague, Czech Republic; 28 Escuela Politecnica Nacional, Quito, Ecuador; 29 Universidad San Francisco de Quito, Quito, Ecuador; 30 Academy of Scientific Research and Technology of the Arab Republic of Egypt, Egyptian Network of High Energy Physics, Cairo, Egypt; 31 Center for High Energy Physics (CHEP-FU), Fayoum University, El-Fayoum, Egypt; 32 National Institute of Chemical Physics and Biophysics, Tallinn, Estonia; 33 Department of Physics, University of Helsinki, Helsinki, Finland; 34 Helsinki Institute of Physics, Helsinki, Finland; 35 Lappeenranta University of Technology, Lappeenranta, Finland; 36 IRFU, CEA, Université Paris-Saclay, Gif-sur-Yvette, France; 37 Laboratoire Leprince-Ringuet, CNRS/IN2P3, Ecole Polytechnique, Institut Polytechnique de Paris, Palaiseau, France; 38 Université de Strasbourg, CNRS, IPHC UMR 7178, Strasbourg, France; 39 Institut de Physique Nucléaire de Lyon, Université de Lyon, Université Claude Bernard Lyon 1, CNRS-IN2P3, Villeurbanne, France; 40 Georgian Technical University, Tbilisi, Georgia; 41 I. Physikalisches Institut, RWTH Aachen University, Aachen, Germany; 42 III. Physikalisches Institut A, RWTH Aachen University, Aachen, Germany; 43 III. Physikalisches Institut B, RWTH Aachen University, Aachen, Germany; 44 Deutsches Elektronen-Synchrotron, Hamburg, Germany; 45 University of Hamburg, Hamburg, Germany; 46 Karlsruher Institut fuer Technologie, Karlsruhe, Germany; 47 Institute of Nuclear and Particle Physics (INPP), NCSR Demokritos, Aghia Paraskevi, Greece; 48 National and Kapodistrian University of Athens, Athens, Greece; 49 National Technical University of Athens, Athens, Greece; 50 University of Ioánnina, Ioannina, Greece; 51 MTA-ELTE Lendület CMS Particle and Nuclear Physics Group, Eötvös Loránd University, Budapest, Hungary; 52 Wigner Research Centre for Physics, Budapest, Hungary; 53 Institute of Nuclear Research ATOMKI, Debrecen, Hungary; 54 Institute of Physics, University of Debrecen, Debrecen, Hungary; 55 Eszterhazy Karoly University, Karoly Robert Campus, Gyongyos, Hungary; 56 Indian Institute of Science (IISc), Bangalore, India; 57 National Institute of Science Education and Research, HBNI, Bhubaneswar, India; 58 Panjab University, Chandigarh, India; 59 University of Delhi, Delhi, India; 60 Saha Institute of Nuclear Physics, HBNI, Kolkata, India; 61 Indian Institute of Technology Madras, Chennai, India; 62 Bhabha Atomic Research Centre, Mumbai, India; 63 Tata Institute of Fundamental Research-A, Mumbai, India; 64 Tata Institute of Fundamental Research-B, Mumbai, India; 65 Indian Institute of Science Education and Research (IISER), Pune, India; 66 Department of Physics, Isfahan University of Technology, Isfahan, Iran; 67 Institute for Research in Fundamental Sciences (IPM), Tehran, Iran; 68 University College Dublin, Dublin, Ireland; 69 INFN Sezione di Bari, Università di Bari, Politecnico di Bari, Bari, Italy; 70 INFN Sezione di Bologna, Università di Bologna, Bologna, Italy; 71 INFN Sezione di Catania, Università di Catania, Catania, Italy; 72 INFN Sezione di Firenze, Università di Firenze, Florence, Italy; 73 INFN Laboratori Nazionali di Frascati, Frascati, Italy; 74 INFN Sezione di Genova, Università di Genova, Genoa, Italy; 75 INFN Sezione di Milano-Bicocca, Università di Milano-Bicocca, Milan, Italy; 76 INFN Sezione di Napoli, Università di Napoli ‘Federico II’, Naples, Italy, Università della Basilicata, Potenza, Italy, Università G. Marconi, Rome, Italy; 77 INFN Sezione di Padova, Università di Padova, Padua, Italy, Università di Trento, Trento, Italy; 78 INFN Sezione di Pavia, Università di Pavia, Pavia, Italy; 79 INFN Sezione di Perugia, Università di Perugia, Perugia, Italy; 80 INFN Sezione di Pisa, Università di Pisa, Scuola Normale Superiore di Pisa, Pisa, Italy, Università di Siena, Siena, Italy; 81 INFN Sezione di Roma, Sapienza Università di Roma, Rome, Italy; 82 INFN Sezione di Torino, Università di Torino, Turin, Italy, Università del Piemonte Orientale, Novara, Italy; 83 INFN Sezione di Trieste, Università di Trieste, Trieste, Italy; 84 Kyungpook National University, Daegu, Korea; 85 Institute for Universe and Elementary Particles, Chonnam National University, Kwangju, Korea; 86 Hanyang University, Seoul, Korea; 87 Korea University, Seoul, Korea; 88 Department of Physics, Kyung Hee University, Seoul, Republic of Korea; 89 Sejong University, Seoul, Korea; 90 Seoul National University, Seoul, Korea; 91 University of Seoul, Seoul, Korea; 92 Department of Physics, Yonsei University, Seoul, Korea; 93 Sungkyunkwan University, Suwon, Korea; 94 College of Engineering and Technology, American University of the Middle East (AUM), Egaila, Kuwait; 95 Riga Technical University, Riga, Latvia; 96 Vilnius University, Vilnius, Lithuania; 97 National Centre for Particle Physics, Universiti Malaya, Kuala Lumpur, Malaysia; 98 Universidad de Sonora (UNISON), Hermosillo, Mexico; 99 Centro de Investigacion y de Estudios Avanzados del IPN, Mexico City, Mexico; 100 Universidad Iberoamericana, Mexico City, Mexico; 101 Benemerita Universidad Autonoma de Puebla, Puebla, Mexico; 102 Universidad Autónoma de San Luis Potosí, San Luis Potosí, Mexico; 103 University of Montenegro, Podgorica, Montenegro; 104 University of Auckland, Auckland, New Zealand; 105 University of Canterbury, Christchurch, New Zealand; 106 National Centre for Physics, Quaid-I-Azam University, Islamabad, Pakistan; 107 Faculty of Computer Science, Electronics and Telecommunications, AGH University of Science and Technology, Kraków, Poland; 108 National Centre for Nuclear Research, Swierk, Poland; 109 Institute of Experimental Physics, Faculty of Physics, University of Warsaw, Warsaw, Poland; 110 Laboratório de Instrumentação e Física Experimental de Partículas, Lisbon, Portugal; 111 Joint Institute for Nuclear Research, Dubna, Russia; 112 Petersburg Nuclear Physics Institute, Gatchina (St. Petersburg), Russia; 113 Institute for Nuclear Research, Moscow, Russia; 114 Institute for Theoretical and Experimental Physics named by A.I. Alikhanov of NRC ‘Kurchatov Institute’, Moscow, Russia; 115 Moscow Institute of Physics and Technology, Moscow, Russia; 116 National Research Nuclear University ‘Moscow Engineering Physics Institute’ (MEPhI), Moscow, Russia; 117 P.N. Lebedev Physical Institute, Moscow, Russia; 118 Skobeltsyn Institute of Nuclear Physics, Lomonosov Moscow State University, Moscow, Russia; 119 Novosibirsk State University (NSU), Novosibirsk, Russia; 120 Institute for High Energy Physics of National Research Centre ‘Kurchatov Institute’, Protvino, Russia; 121 National Research Tomsk Polytechnic University, Tomsk, Russia; 122 Tomsk State University, Tomsk, Russia; 123 Faculty of Physics and VINCA Institute of Nuclear Sciences, University of Belgrade, Belgrade, Serbia; 124 Centro de Investigaciones Energéticas Medioambientales y Tecnológicas (CIEMAT), Madrid, Spain; 125 Universidad Autónoma de Madrid, Madrid, Spain; 126 Instituto Universitario de Ciencias y Tecnologías Espaciales de Asturias (ICTEA), Universidad de Oviedo, Oviedo, Spain; 127 Instituto de Física de Cantabria (IFCA), CSIC-Universidad de Cantabria, Santander, Spain; 128 University of Colombo, Colombo, Sri Lanka; 129 Department of Physics, University of Ruhuna, Matara, Sri Lanka; 130 CERN, European Organization for Nuclear Research, Geneva, Switzerland; 131 Paul Scherrer Institut, Villigen, Switzerland; 132 ETH Zurich-Institute for Particle Physics and Astrophysics (IPA), Zurich, Switzerland; 133 Universität Zürich, Zurich, Switzerland; 134 National Central University, Chung-Li, Taiwan; 135 National Taiwan University (NTU), Taipei, Taiwan; 136 Department of Physics, Faculty of Science, Chulalongkorn University, Bangkok, Thailand; 137 Physics Department, Science and Art Faculty, Çukurova University, Adana, Turkey; 138 Physics Department, Middle East Technical University, Ankara, Turkey; 139 Bogazici University, Istanbul, Turkey; 140 Istanbul Technical University, Istanbul, Turkey; 141 Istanbul University, Istanbul, Turkey; 142 Institute for Scintillation Materials of National Academy of Science of Ukraine, Kharkov, Ukraine; 143 National Scientific Center, Kharkov Institute of Physics and Technology, Kharkov, Ukraine; 144 University of Bristol, Bristol, UK; 145 Rutherford Appleton Laboratory, Didcot, UK; 146 Imperial College, London, UK; 147 Brunel University, Uxbridge, UK; 148 Baylor University, Waco, USA; 149 Catholic University of America, Washington, DC, USA; 150 The University of Alabama, Tuscaloosa, USA; 151 Boston University, Boston, USA; 152 Brown University, Providence, USA; 153 University of California, Davis, Davis, USA; 154 University of California, Los Angeles, USA; 155 University of California, Riverside, Riverside, USA; 156 University of California, San Diego, La Jolla, USA; 157 Department of Physics, University of California, Santa Barbara, Santa Barbara, USA; 158 California Institute of Technology, Pasadena, USA; 159 Carnegie Mellon University, Pittsburgh, USA; 160 University of Colorado Boulder, Boulder, USA; 161 Cornell University, Ithaca, USA; 162 Fermi National Accelerator Laboratory, Batavia, USA; 163 University of Florida, Gainesville, USA; 164 Florida State University, Tallahassee, USA; 165 Florida Institute of Technology, Melbourne, USA; 166 University of Illinois at Chicago (UIC), Chicago, USA; 167 The University of Iowa, Iowa City, USA; 168 Johns Hopkins University, Baltimore, USA; 169 The University of Kansas, Lawrence, USA; 170 Kansas State University, Manhattan, USA; 171 Lawrence Livermore National Laboratory, Livermore, USA; 172 University of Maryland, College Park, USA; 173 Massachusetts Institute of Technology, Cambridge, USA; 174 University of Minnesota, Minneapolis, USA; 175 University of Mississippi, Oxford, USA; 176 University of Nebraska-Lincoln, Lincoln, USA; 177 State University of New York at Buffalo, Buffalo, USA; 178 Northeastern University, Boston, USA; 179 Northwestern University, Evanston, USA; 180 University of Notre Dame, Notre Dame, USA; 181 The Ohio State University, Columbus, USA; 182 Princeton University, Princeton, USA; 183 University of Puerto Rico, Mayagüez, USA; 184 Purdue University, West Lafayette, USA; 185 Purdue University Northwest, Hammond, USA; 186 Rice University, Houston, USA; 187 University of Rochester, Rochester, USA; 188 Rutgers, The State University of New Jersey, Piscataway, USA; 189 University of Tennessee, Knoxville, USA; 190 Texas A&M University, College Station, USA; 191 Texas Tech University, Lubbock, USA; 192 Vanderbilt University, Nashville, USA; 193 University of Virginia, Charlottesville, USA; 194 Wayne State University, Detroit, USA; 195 University of Wisconsin-Madison, Madison, WI USA; 196 Vienna University of Technology, Vienna, Austria; 197 Institute of Basic and Applied Sciences, Faculty of Engineering, Arab Academy for Science, Technology and Maritime Transport, Alexandria, Egypt; 198 Université Libre de Bruxelles, Brussels, Belgium; 199 IRFU, CEA, Université Paris-Saclay, Gif-sur-Yvette, France; 200 Universidade Estadual de Campinas, Campinas, Brazil; 201 Federal University of Rio Grande do Sul, Porto Alegre, Brazil; 202 UFMS, Nova Andradina, Brazil; 203 Universidade Federal de Pelotas, Pelotas, Brazil; 204 Department of Physics, Nanjing Normal University, Nanjing, China; 205 The University of Iowa, Iowa City, USA; 206 University of Chinese Academy of Sciences, Beijing, China; 207 Institute for Theoretical and Experimental Physics named by A.I. Alikhanov of NRC ‘Kurchatov Institute’, Moscow, Russia; 208 Joint Institute for Nuclear Research, Dubna, Russia; 209 Ain Shams University, Cairo, Egypt; 210 Suez University, Suez, Egypt; 211 British University in Egypt, Cairo, Egypt; 212 Purdue University, West Lafayette, USA; 213 Université de Haute Alsace, Mulhouse, France; 214 Erzincan Binali Yildirim University, Erzincan, Turkey; 215 CERN, European Organization for Nuclear Research, Geneva, Switzerland; 216 III. Physikalisches Institut A, RWTH Aachen University, Aachen, Germany; 217 University of Hamburg, Hamburg, Germany; 218 Department of Physics, Isfahan University of Technology, Isfahan, Iran; 219 Brandenburg University of Technology, Cottbus, Germany; 220 Skobeltsyn Institute of Nuclear Physics, Lomonosov Moscow State University, Moscow, Russia; 221 Institute of Physics, University of Debrecen, Debrecen, Hungary; 222 Physics Department, Faculty of Science, Assiut University, Assiut, Egypt; 223 Eszterhazy Karoly University, Karoly Robert Campus, Gyongyos, Hungary; 224 Institute of Nuclear Research ATOMKI, Debrecen, Hungary; 225 MTA-ELTE Lendület CMS Particle and Nuclear Physics Group, Eötvös Loránd University, Budapest, Hungary; 226 Wigner Research Centre for Physics, Budapest, Hungary; 227 IIT Bhubaneswar, Bhubaneswar, India; 228 Institute of Physics, Bhubaneswar, India; 229 G.H.G. Khalsa College, Punjab, India; 230 Shoolini University, Solan, India; 231 University of Hyderabad, Hyderabad, India; 232 University of Visva-Bharati, Santiniketan, India; 233 Indian Institute of Technology (IIT), Mumbai, India; 234 Deutsches Elektronen-Synchrotron, Hamburg, Germany; 235 Sharif University of Technology, Tehran, Iran; 236 Department of Physics, University of Science and Technology of Mazandaran, Behshahr, Iran; 237 INFN Sezione di Bari, Università di Bari, Politecnico di Bari, Bari, Italy; 238 Italian National Agency for New Technologies, Energy and Sustainable Economic Development, Bologna, Italy; 239 Centro Siciliano di Fisica Nucleare e di Struttura Della Materia, Catania, Italy; 240 Università di Napoli ‘Federico II’, Naples, Italy; 241 Riga Technical University, Riga, Latvia; 242 Consejo Nacional de Ciencia y Tecnología, Mexico City, Mexico; 243 Institute for Nuclear Research, Moscow, Russia; 244 National Research Nuclear University ‘Moscow Engineering Physics Institute’ (MEPhI), Moscow, Russia; 245 St. Petersburg State Polytechnical University, St. Petersburg, Russia; 246 University of Florida, Gainesville, USA; 247 Imperial College, London, UK; 248 P.N. Lebedev Physical Institute, Moscow, Russia; 249 California Institute of Technology, Pasadena, USA; 250 Budker Institute of Nuclear Physics, Novosibirsk, Russia; 251 Faculty of Physics, University of Belgrade, Belgrade, Serbia; 252 Trincomalee Campus, Eastern University, Sri Lanka, Nilaveli, Sri Lanka; 253 INFN Sezione di Pavia, Università di Pavia, Pavia, Italy; 254 National and Kapodistrian University of Athens, Athens, Greece; 255 Universität Zürich, Zurich, Switzerland; 256 Ecole Polytechnique Fédérale Lausanne, Lausanne, Switzerland; 257 Stefan Meyer Institute for Subatomic Physics, Vienna, Austria; 258 Laboratoire d’Annecy-le-Vieux de Physique des Particules, IN2P3-CNRS, Annecy-le-Vieux, France; 259 Gaziosmanpasa University, Tokat, Turkey; 260 Şırnak University, Sirnak, Turkey; 261 Department of Physics, Tsinghua University, Beijing, China; 262 Research Center of Experimental Health Science, Near East University, Nicosia, Turkey; 263 Beykent University, Istanbul, Turkey; 264 Application and Research Center for Advanced Studies (App. and Res. Cent. for Advanced Studies), Istanbul Aydin University, Istanbul, Turkey; 265 Mersin University, Mersin, Turkey; 266 Adiyaman University, Adiyaman, Turkey; 267 Tarsus University, Mersin, Turkey; 268 Ozyegin University, Istanbul, Turkey; 269 Izmir Institute of Technology, Izmir, Turkey; 270 Necmettin Erbakan University, Konya, Turkey; 271 Bozok Universitetesi Rektörlügü, Yozgat, Turkey; 272 Marmara University, Istanbul, Turkey; 273 Milli Savunma University, Istanbul, Turkey; 274 Kafkas University, Kars, Turkey; 275 Istanbul Bilgi University, Istanbul, Turkey; 276 Hacettepe University, Ankara, Turkey; 277 Vrije Universiteit Brussel, Brussels, Belgium; 278 School of Physics and Astronomy, University of Southampton, Southampton, UK; 279 IPPP Durham University, Durham, UK; 280 Faculty of Science, Monash University, Clayton, Australia; 281 Bethel University, St. Paul, Minneapolis, USA, St. Paul, USA; 282 Karamanoğlu Mehmetbey University, Karaman, Turkey; 283 Bingol University, Bingol, Turkey; 284 Georgian Technical University, Tbilisi, Georgia; 285 Sinop University, Sinop, Turkey; 286 Mimar Sinan University, Istanbul, Turkey; 287 Texas A&M University at Qatar, Doha, Qatar; 288 Kyungpook National University, Daegu, Korea; 289 CERN, 1211 Geneva 23, Switzerland

## Abstract

A search is presented for a heavy vector resonance decaying into a ${\mathrm{Z}}_{\mathrm{}}^{\mathrm{}}$ boson and the standard model Higgs boson, where the ${\mathrm{Z}}_{\mathrm{}}^{\mathrm{}}$ boson is identified through its leptonic decays to electrons, muons, or neutrinos, and the Higgs boson is identified through its hadronic decays. The search is performed in a Lorentz-boosted regime and is based on data collected from 2016 to 2018 at the CERN LHC, corresponding to an integrated luminosity of 137$\,\text {fb}^{-1}$. Upper limits are derived on the production of a narrow heavy resonance ${\mathrm{{{\mathrm{Z}}_{\mathrm{}}^{\mathrm{}}}}}_{\mathrm{}}^{\mathrm{\prime }}$, and a mass below 3.5 and 3.7$\,\text {Te}\text {V}$ is excluded at 95% confidence level in models where the heavy vector boson couples predominantly to fermions and to bosons, respectively. These are the most stringent limits placed on the Heavy Vector Triplet ${\mathrm{{{\mathrm{Z}}_{\mathrm{}}^{\mathrm{}}}}}_{\mathrm{}}^{\mathrm{\prime }}$ model to date. If the heavy vector boson couples exclusively to standard model bosons, upper limits on the product of the cross section and branching fraction are set between 23 and 0.3$\,\text {fb}$ for a ${\mathrm{{{\mathrm{Z}}_{\mathrm{}}^{\mathrm{}}}}}_{\mathrm{}}^{\mathrm{\prime }}$ mass between 0.8 and 4.6$\,\text {Te}\text {V}$, respectively. This is the first limit set on a heavy vector boson coupling exclusively to standard model bosons in its production and decay.

## Introduction

The discovery of a Higgs boson (${\mathrm{H}}_{\mathrm{}}^{\mathrm{}}$) [1–3] by the ATLAS and CMS Collaborations at the CERN LHC, with properties consistent with expectations from the standard model (SM) of particle physics, has emphasized the hierarchy problem of the SM. In the SM, the measured ${\mathrm{H}}_{\mathrm{}}^{\mathrm{}}$ mass of 125$\,\text {Ge}\text {V}$  [4, 5], given its fundamental scalar nature [6, 7], requires extreme fine tuning of quantum corrections, suggesting that the SM may be incomplete. Many different exotic models, such as the little Higgs [8–10] and composite Higgs [11–13] models, predict the existence of new resonances decaying to a vector boson (${{\mathrm{V}}_{\mathrm{}}^{\mathrm{}}} = {{\mathrm{W}}_{\mathrm{}}^{\mathrm{}}}, {{\mathrm{Z}}_{\mathrm{}}^{\mathrm{}}}$) and a Higgs boson [14–18].

Heavy vector triplet (HVT) models [19] introduce new heavy vector bosons (${\mathrm{{{\mathrm{W}}_{\mathrm{}}^{\mathrm{}}}}}_{\mathrm{}}^{\mathrm{\prime }}$, ${\mathrm{{{\mathrm{Z}}_{\mathrm{}}^{\mathrm{}}}}}_{\mathrm{}}^{\mathrm{\prime }}$) that couple to the Higgs and SM gauge bosons with the parameters $c_\text {H}$ and $g_\text {V}$, and to the fermions via the combination $(g^2/g_\text {V}) c_\text {F}$, where $c_\text {F}$ is the fermion coupling and *g* is the SM $\text {SU}(2)_\text {L}$ gauge coupling. The HVT couplings are expected to be of order unity in most models. Three benchmark models, denoted as models A, B, and C are considered in this paper.

In model A, the coupling strengths to fermions and gauge bosons are comparable and the heavy resonances decay predominantly to fermions, as is the case in some extensions of the SM gauge group [20]. In model B, the fermionic couplings are suppressed, as in composite Higgs models. In model C, the fermionic couplings are set to zero, so the resonances are produced only through vector boson fusion (VBF) and decay exclusively to a pair of SM bosons. The parameters used for model A are $g_\text {V} = 1$, $c_\text {H} = -0.556$, and $c_\text {F} = -1.316$; for model B, $g_\text {V} = 3$, $c_\text {H} = -0.976$, and $c_\text {F} = 1.024$; and for model C, $g_\text {V} = 1$, $c_\text {H} = 1$, $c_\text {F} = 0$.

Previous searches for a heavy resonance decaying to a Higgs boson and a vector boson have been carried out at $\sqrt{s}= 13\,\text {Te}\text {V}$ in the semileptonic final state [14, 15, 21] and in the fully hadronic final state [22–24] by the CMS and ATLAS Collaborations. The most stringent lower limit on the ${\mathrm{{{\mathrm{Z}}_{\mathrm{}}^{\mathrm{}}}}}_{\mathrm{}}^{\mathrm{\prime }}$ mass at 95% confidence level using the semileptonic (fully hadronic) final state is 2.65 (2.2)$\,\text {Te}\text {V}$ in HVT model A and 2.83 (2.65)$\,\text {Te}\text {V}$ in HVT model B [15, 24].

**Fig. 1:**
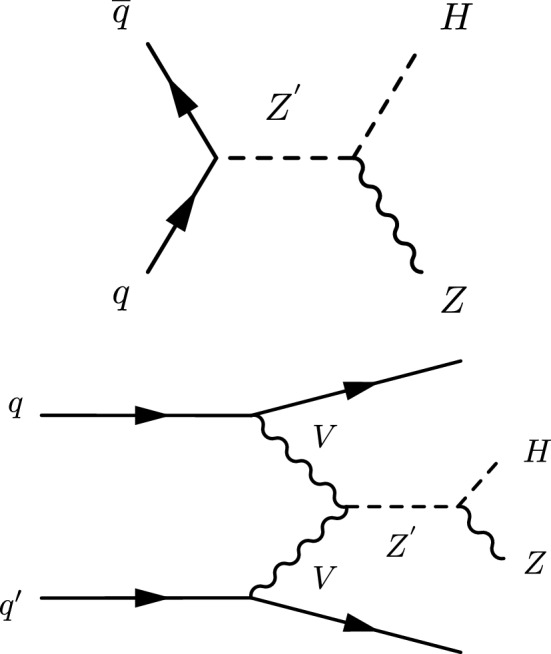
The leading order Feynman diagrams of the heavy resonance ${\mathrm{{{\mathrm{Z}}_{\mathrm{}}^{\mathrm{}}}}}_{\mathrm{}}^{\mathrm{\prime }}$ production through ${{\mathrm{q}}_{\mathrm{}}^{\mathrm{}}} {{\overline{{{{\mathrm{q}}_{\mathrm{}}^{\mathrm{}}}}}}}$ annihilation (upper) and vector boson fusion (lower), decaying to a ${\mathrm{Z}}_{\mathrm{}}^{\mathrm{}}$ boson (${\mathrm{Z}}_{\mathrm{}}^{\mathrm{}}$) and a Higgs boson (${\mathrm{H}}_{\mathrm{}}^{\mathrm{}}$)

This paper describes a search for a heavy resonance (denoted as ${\mathrm{X}}_{\mathrm{}}^{\mathrm{}}$ for the reconstructed quantity and ${\mathrm{{{\mathrm{Z}}_{\mathrm{}}^{\mathrm{}}}}}_{\mathrm{}}^{\mathrm{\prime }}$ for the particle predicted by the theory) decaying to a ${\mathrm{Z}}_{\mathrm{}}^{\mathrm{}}$ boson and a Higgs boson. The ${\mathrm{Z}}_{\mathrm{}}^{\mathrm{}}$ boson is identified via a pair of electrons or muons, or a large amount of missing transverse momentum (${\vec p}_{\mathrm {T}}^{\text {miss}}$) measured in the detector due to the presence of at least two neutrinos. The Higgs boson is identified via its hadronic decays, either directly to a pair of heavy quarks, or via cascade decays dominated by ${{\mathrm{W}}_{\mathrm{}}^{\mathrm{}}} {{\mathrm{W}}_{\mathrm{}}^{\mathrm{}}}$ and ${{\mathrm{Z}}_{\mathrm{}}^{\mathrm{}}} {{\mathrm{Z}}_{\mathrm{}}^{\mathrm{}}}$. We explore the regime where the Higgs boson has a large Lorentz boost and is reconstructed as a single, large-radius jet, referred to as $j_{{{\mathrm{H}}_{\mathrm{}}^{\mathrm{}}}}$, with characteristic substructure and identified via its mass and possible presence of ${\mathrm{b}}_{\mathrm{}}^{\mathrm{}}$ quark subjets. If a heavy resonance couples exclusively to the SM bosons, it can be produced dominantly through VBF. Dedicated categories are defined in order to enhance the sensitivity to this production mode, exploiting the presence of two jets with large transverse momenta ($p_{\mathrm {T}}$) in the forward region of the detector, which are remnants of the initial-state quarks participating in the VBF interaction. The Feynman diagrams for the signal processes are depicted in Fig. 1.

The search is performed by examining the distribution of the reconstructed mass ($m_{{{\mathrm{X}}_{\mathrm{}}^{\mathrm{}}}}$) or transverse mass ($m_{{{\mathrm{X}}_{\mathrm{}}^{\mathrm{}}}}^{\text {T}}$) of the heavy resonance for a localized excess of events. The main background normalization is determined from data in sideband regions (SBs) of the $j_{{{\mathrm{H}}_{\mathrm{}}^{\mathrm{}}}}$ mass distribution, and extrapolated to the signal region (SR) through analytical functions derived from simulation.

## The CMS detector

The CMS detector features a silicon pixel and strip tracker, a lead tungstate crystal electromagnetic calorimeter (ECAL), and a brass and scintillator hadron calorimeter, each composed of a barrel and two endcap sections. These detectors reside within a superconducting solenoid, which provides a magnetic field of 3.8$\,\text {T}$. Forward calorimeters extend the pseudorapidity $\eta$ coverage up to $|\eta | < 5.2$. Muons are measured in gas-ionization detectors embedded in the steel flux-return yoke outside the solenoid. A detailed description of the CMS detector, together with a definition of the coordinate system and the kinematic variables, can be found in Ref. [25].

Events of interest are selected using a two-tiered trigger system [26]. The first level, composed of custom hardware processors, uses information from the calorimeters and muon detectors to select events at a rate of around 100$\,\text {kHz}$ within a fixed time interval of about 4$\,\upmu \text {s}$. The second level, known as the high-level trigger (HLT), consists of a farm of processors running a version of the full event reconstruction software optimized for fast processing, and reduces the event rate to around 1$\,\text {kHz}$ before data storage.

## Data and simulated samples

The data samples used in this search were collected during the period 2016–2018, with the CMS detector at the LHC in proton–proton (${{\mathrm{p}}_{\mathrm{}}^{\mathrm{}}} {{\mathrm{p}}_{\mathrm{}}^{\mathrm{}}}$) collisions at a center-of-mass energy of 13$\,\text {Te}\text {V}$, resulting in a combined integrated luminosity of 137$\,\text {fb}^{-1}$.

The signal samples are generated at leading order (LO) through ${{\mathrm{q}}_{\mathrm{}}^{\mathrm{}}} {{\overline{{{{\mathrm{q}}_{\mathrm{}}^{\mathrm{}}}}}}}$ annihilation, taking the cross sections from HVT models A and B [19], or through VBF with the cross section from HVT model C, using the MadGraph 5_amc@nlo 2.4.2 [27] generator and the MLM matching scheme [28]. Different hypotheses for the heavy resonance mass in the range of 800–5000$\,\text {Ge}\text {V}$ are considered, with the natural width of the resonance being negligible compared to the 4% detector resolution (the narrow-width approximation). The heavy resonance is forced to decay to a ${\mathrm{Z}}_{\mathrm{}}^{\mathrm{}}$ boson and a Higgs boson, with the former decaying into a pair of charged leptons ($\ell = {{\mathrm{e}}_{\mathrm{}}^{\mathrm{}}}$ or ${\upmu {}{}}$) or neutrinos, including cascade decays involving tau leptons. There is no restriction on the decay channels for the Higgs boson and its decay particles, which decay according to the SM branching fractions.

The SM background for this search is dominated by ${{\mathrm{V}}_{\mathrm{}}^{\mathrm{}}} \text {+jets}$ production, with the ${\mathrm{V}}_{\mathrm{}}^{\mathrm{}}$ boson decaying as ${{\mathrm{Z}}_{\mathrm{}}^{\mathrm{}}} \rightarrow \nu \nu$, ${{\mathrm{Z}}_{\mathrm{}}^{\mathrm{}}} \rightarrow {{\mathrm{e}}_{\mathrm{}}^{\mathrm{}}} \bar{{{\mathrm{e}}_{\mathrm{}}^{\mathrm{}}}},{\upmu {}{}} \bar{{\upmu {}{}}},{\uptau {}{}} \bar{{\uptau {}{}}}$, or ${{\mathrm{W}}_{\mathrm{}}^{\mathrm{}}} \rightarrow {{\mathrm{e}}_{\mathrm{}}^{\mathrm{}}} \nu ,{\upmu {}{}} \nu ,{\uptau {}{}} \nu$. The ${{\mathrm{V}}_{\mathrm{}}^{\mathrm{}}} \text {+jets}$ background sample is produced with the MadGraph 5_amc@nlo generator at LO. The sample is further normalized to account for next-to-LO (NLO) in electroweak (EW) and next-to-NLO (NNLO) in quantum chromodynamics (QCD) corrections to the cross section from Ref. [29]. The top quark pair (${\mathrm{t}}_{\mathrm{}}^{\mathrm{}}$ $\overline{{{{\mathrm{t}}_{\mathrm{}}^{\mathrm{}}}}}$) and single top quark *t*-channel and ${{\mathrm{t}}_{\mathrm{}}^{\mathrm{}}}{{\mathrm{W}}_{\mathrm{}}^{\mathrm{}}}$ production are generated at NLO in QCD with the powheg 2.0 generator [30–35]. The ${\mathrm{t}}_{\mathrm{}}^{\mathrm{}}$ $\overline{{{{\mathrm{t}}_{\mathrm{}}^{\mathrm{}}}}}$ samples are normalized to the cross section computed with Top++ 2.0 [36] at NNLO in QCD with next-to-next-to-leading logarithmic soft gluon resummation accuracy. The single top quark *s*-channel, ${{\mathrm{V}}_{\mathrm{}}^{\mathrm{}}} {{\mathrm{V}}_{\mathrm{}}^{\mathrm{}}}$, and ${{\mathrm{V}}_{\mathrm{}}^{\mathrm{}}} {{\mathrm{H}}_{\mathrm{}}^{\mathrm{}}}$ samples are simulated at NLO in QCD with the MadGraph 5_amc@nlo generator.

The NNPDF 3.0 [37] set of parton distribution functions (PDF) is used to simulate the hard process in all simulated samples for the 2016 data and the NNPDF 3.1 [38] set is used for 2017 and 2018. Parton showering and hadronization processes are performed with pythia 8.226 [39] with the CUETP8M1 [40, 41] underlying event tune for 2016, and pythia 8.230 with the CP5 [42] event tune for 2017 and 2018. The CUETP8M2 underlying event tune [43] is used to simulate ${\mathrm{t}}_{\mathrm{}}^{\mathrm{}}$ $\overline{{{{\mathrm{t}}_{\mathrm{}}^{\mathrm{}}}}}$ production for 2016 samples. The CMS detector response simulation is performed with Geant4  [44]. Simulated samples are reconstructed with the same software as used for collision data. The data samples contain additional ${{\mathrm{p}}_{\mathrm{}}^{\mathrm{}}} {{\mathrm{p}}_{\mathrm{}}^{\mathrm{}}}$ interactions in the same or nearby bunch crossings (pileup). The simulated pileup description is reweighted to match the distribution of the pileup multiplicity measured in data.

## Event reconstruction

Events in the CMS detector are reconstructed using the particle-flow (PF) algorithm [45], which combines information from all subdetectors in order to reconstruct stable particles (muons, electrons, photons, neutral and charged hadrons). Jets are reconstructed from PF candidates clustered with the anti-$k_{\mathrm {T}}$ algorithm [46], with a distance parameter of 0.4 (AK4 jets) or 0.8 (AK8 jets), using the FastJet 3.0 package [47, 48]. Several vertices are reconstructed per bunch crossing. The candidate vertex with the largest value of summed physics-object $p_{\mathrm {T}} ^2$ is taken to be the primary ${{\mathrm{p}}_{\mathrm{}}^{\mathrm{}}} {{\mathrm{p}}_{\mathrm{}}^{\mathrm{}}}$ interaction vertex. Here the physics objects are the AK4 jets, clustered using the jet finding algorithms with the tracks assigned to candidate vertices as inputs, and the associated ${\vec p}_{\mathrm {T}}^{\text {miss}}$ taken as the negative vector $p_{\mathrm {T}}$ sum of those jets. Two different methods to remove contributions from pileup are used: for the AK4 jets, pileup is accounted for via the charged-hadron subtraction algorithm [49] in conjunction with the jet area method [50], while for the AK8 jets the pileup-per-particle identification algorithm [51] is employed. The jet energy resolution, after the application of corrections to the jet energy, is 4% at 1$\,\text {Te}\text {V}$  [52]. For the AK4 jets, $p_{\mathrm {T}} > 30\,\text {Ge}\text {V}$ and $|\eta | < 2.4$ are required, and jets within a cone of $\varDelta R(j,\ell )=\sqrt{\smash [b]{\varDelta \eta (j,\ell )^2+\varDelta \phi (j,\ell )^2}}>0.4$ around isolated leptons are removed, where $\phi$ is the azimuthal angle. The AK8 jets must satisfy $p_{\mathrm {T}} > 200$ $\,\text {Ge}\text {V}$ and $|\eta | < 2.4$. The vector ${\vec p}_{\mathrm {T}}^{\text {miss}}$ is computed as the negative vector $p_{\mathrm {T}}$ sum of all the PF candidates in an event. The ${\vec p}_{\mathrm {T}}^{\text {miss}}$ is corrected for adjustments to the energy scale of the reconstructed AK4 jets in the event, and its magnitude is denoted as $p_{\mathrm {T}} ^\text {miss}$  [53]. The observable $H_{\mathrm {T}}^{\text {miss}}$ is defined as the magnitude of the vector $p_{\mathrm {T}}$ sum of all AK4 jets with $p_{\mathrm {T}} > 30\,\text {Ge}\text {V}$ and $|\eta | < 3.0$.

For each AK8 jet a groomed jet mass ($m_j$) is calculated, after applying a modified mass-drop algorithm [54, 55]. The mass-drop algorithm used here is known as the soft-drop algorithm [56], with parameters $\beta =0$, $z_\text {cut}=0.1$, and $R_0 = 0.8$. Subjets are obtained by reverting the last step of the jet clustering and selecting the two with the highest $p_{\mathrm {T}}$. The groomed jet mass is calibrated in a ${\mathrm{t}}_{\mathrm{}}^{\mathrm{}}$ $\overline{{{{\mathrm{t}}_{\mathrm{}}^{\mathrm{}}}}}$ sample enriched in hadronically decaying ${\mathrm{W}}_{\mathrm{}}^{\mathrm{}}$ bosons [57].

The identification of jets that originate from ${\mathrm{b}}_{\mathrm{}}^{\mathrm{}}$ quarks is performed with the DeepCSV algorithm [58], which is based on a deep neural network with information on tracks and secondary vertices associated with the jet as inputs. The DeepCSV algorithm is applied to AK4 jets and the two highest $p_{\mathrm {T}}$ AK8 subjets. A jet is considered as ${\mathrm{b}}_{\mathrm{}}^{\mathrm{}}$ tagged if the output discriminator value is larger than a defined threshold, corresponding to a 75% ${\mathrm{b}}_{\mathrm{}}^{\mathrm{}}$ tagging efficiency with a probability for mistagging jets originating from the hadronization of gluons or ${\mathrm{u}}_{\mathrm{}}^{\mathrm{}}$/${\mathrm{d}}_{\mathrm{}}^{\mathrm{}}$/${\mathrm{s}}_{\mathrm{}}^{\mathrm{}}$ quarks of about 3%. The simulated samples are reweighted to account for small differences in the ${\mathrm{b}}_{\mathrm{}}^{\mathrm{}}$ tagging efficiency from values obtained in data.

Electrons are reconstructed from ECAL energy deposits in the range $|\eta |<2.5$ that are matched to tracks reconstructed in the silicon tracker. The electrons are identified taking into account the distribution of energy deposited along the electron trajectory, the direction and momentum of the track, and its compatibility with the primary vertex [59]. Electrons are required to pass an isolation requirement. The isolation is defined as the $p_{\mathrm {T}}$ sum of all particles within a cone of $\varDelta R = 0.3$ around the electron track, after the contributions from the electron itself, other nearby electrons, and pileup are removed. The electron reconstruction efficiency is larger than 88%.

Muons are reconstructed within the acceptance of $|\eta | < 2.4$ by matching tracks in the silicon tracker and charge deposits (hits) in the muon spectrometer. Muon candidates are identified via selection criteria based on the compatibility of tracks reconstructed from only silicon tracker information with tracks reconstructed from a combination of the hits in both the tracker and muon detector. Additional requirements are based on the compatibility of the trajectory with the primary vertex, and on the number of hits observed in the tracker and muon systems. Muons are required to be isolated by imposing a limit on the $p_{\mathrm {T}}$ sum of all the reconstructed tracks within a cone $\varDelta R = 0.4$ around the muon direction, excluding the tracks attributed to muons, divided by the muon $p_{\mathrm {T}}$. The efficiency to reconstruct and identify muons is larger than 96% [60].

Hadronically decaying $\tau$ leptons (${\uptau {}{}} _\mathrm {h}$) are reconstructed by combining one or three charged particles with up to two neutral pion candidates. The selection criteria for the ${\uptau {}{}} _\mathrm {h}$ candidates, which are used to veto various backgrounds, are $p_{\mathrm {T}} > 18\,\text {Ge}\text {V}$, $|\eta | < 2.3$, and $\varDelta R > 0.4$, where $\varDelta R$ is a candidate’s separation from isolated electrons and muons in the event [61].

## Event selection

Events are divided into categories depending on the number and flavor of the reconstructed leptons, the number of ${\mathrm{b}}_{\mathrm{}}^{\mathrm{}}$-tagged subjets of the Higgs candidate jet ($j_{{{\mathrm{H}}_{\mathrm{}}^{\mathrm{}}}}$), and the presence of forward jets consistent with originating from VBF processes. In total, 12 categories are defined and listed in Table 1.

**Table 1:** List of the 12 event categories used in the analysis

$0\ell$, 2${\mathrm{b}}_{\mathrm{}}^{\mathrm{}}$ tag, non-VBF	$0\ell$, 2${\mathrm{b}}_{\mathrm{}}^{\mathrm{}}$ tag, VBF
$2{{\mathrm{e}}_{\mathrm{}}^{\mathrm{}}}$, 2${\mathrm{b}}_{\mathrm{}}^{\mathrm{}}$ tag, non-VBF	$2{{\mathrm{e}}_{\mathrm{}}^{\mathrm{}}}$, 2${\mathrm{b}}_{\mathrm{}}^{\mathrm{}}$ tag, VBF
$2{\upmu {}{}}$, 2${\mathrm{b}}_{\mathrm{}}^{\mathrm{}}$ tag, non-VBF	$2{\upmu {}{}}$, 2${\mathrm{b}}_{\mathrm{}}^{\mathrm{}}$ tag, VBF
$0\ell$, $\le$1${\mathrm{b}}_{\mathrm{}}^{\mathrm{}}$ tag, non-VBF	$0\ell$, $\le$1${\mathrm{b}}_{\mathrm{}}^{\mathrm{}}$ tag, VBF
$2{{\mathrm{e}}_{\mathrm{}}^{\mathrm{}}}$, $\le$1${\mathrm{b}}_{\mathrm{}}^{\mathrm{}}$ tag, non-VBF	$2{{\mathrm{e}}_{\mathrm{}}^{\mathrm{}}}$, $\le$1${\mathrm{b}}_{\mathrm{}}^{\mathrm{}}$ tag, VBF
$2{\upmu {}{}}$, $\le$1${\mathrm{b}}_{\mathrm{}}^{\mathrm{}}$ tag, non-VBF	$2{\upmu {}{}}$, $\le$1${\mathrm{b}}_{\mathrm{}}^{\mathrm{}}$ tag, VBF

The highest $p_{\mathrm {T}}$ AK8 jet in the event is assigned to $j_{{{\mathrm{H}}_{\mathrm{}}^{\mathrm{}}}}$, and is required to have a transverse momentum $p_{\mathrm {T}} ^{{{\mathrm{H}}_{\mathrm{}}^{\mathrm{}}}}> 200\,\text {Ge}\text {V}$ and $|\eta | < 2.4$. This is the correct jet choice in 96% of the simulated signal events. The minimal separation between $j_{{{\mathrm{H}}_{\mathrm{}}^{\mathrm{}}}}$ and isolated leptons from the ${\mathrm{Z}}_{\mathrm{}}^{\mathrm{}}$ boson decay is required to satisfy $\varDelta R(j_{{{\mathrm{H}}_{\mathrm{}}^{\mathrm{}}}},\ell ) > 0.8$. The mass of the $j_{{{\mathrm{H}}_{\mathrm{}}^{\mathrm{}}}}$ jet is required to be compatible with the ${\mathrm{H}}_{\mathrm{}}^{\mathrm{}}$ mass ($105<m_{j_{{{\mathrm{H}}_{\mathrm{}}^{\mathrm{}}}}} <135\,\text {Ge}\text {V}$). It can have 0, 1, or 2 subjets that pass the ${\mathrm{b}}_{\mathrm{}}^{\mathrm{}}$ tagging selection. If both subjets are ${\mathrm{b}}_{\mathrm{}}^{\mathrm{}}$ tagged, the event belongs to the 2${\mathrm{b}}_{\mathrm{}}^{\mathrm{}}$ tag category, otherwise it is assigned to the $\le$1${\mathrm{b}}_{\mathrm{}}^{\mathrm{}}$ tag category.

The $0\ell$ categories require $p_{\mathrm {T}} ^\text {miss} > 250\,\text {Ge}\text {V}$, originating from the Lorentz-boosted ${\mathrm{Z}}_{\mathrm{}}^{\mathrm{}}$ boson decaying to two neutrinos, which leave the detector unobserved. Data are collected using trigger selections that require $p_{\mathrm {T}} ^\text {miss} > 110\,\text {Ge}\text {V}$, calculated with or without considering muons, or $H_{\mathrm {T}}^{\text {miss}} > 110\,\text {Ge}\text {V}$. The minimal azimuthal angular separation between all AK4 jets and the ${\vec p}_{\mathrm {T}}^{\text {miss}}$ vector has to satisfy $\varDelta \phi (j,{\vec p}_{\mathrm {T}}^{\text {miss}}) > 0.5$ in order to suppress multijet production. The azimuthal angular separation between $j_{{{\mathrm{H}}_{\mathrm{}}^{\mathrm{}}}}$ and ${\vec p}_{\mathrm {T}}^{\text {miss}}$ must satisfy $\varDelta \phi (j_{{{\mathrm{H}}_{\mathrm{}}^{\mathrm{}}}},{\vec p}_{\mathrm {T}}^{\text {miss}}) > 2$. Events arising from detector noise are removed by requiring that the fractional contribution of charged hadron candidates to the ${\mathrm{H}}_{\mathrm{}}^{\mathrm{}}$ momentum be larger than 0.1, and the ratio $p_{\mathrm {T}} ^\text {miss}/p_{\mathrm {T}} ^{{\mathrm{H}}_{\mathrm{}}^{\mathrm{}}}$ be larger than 0.6. Events with isolated leptons with $p_{\mathrm {T}} > 10\,\text {Ge}\text {V}$ or hadronically decaying $\tau$ leptons with $p_{\mathrm {T}} > 18$ $\,\text {Ge}\text {V}$ are removed in order to reduce the contribution from other SM processes. The ${\mathrm{t}}_{\mathrm{}}^{\mathrm{}}$ $\overline{{{{\mathrm{t}}_{\mathrm{}}^{\mathrm{}}}}}$ contribution is reduced by removing events with an additional ${\mathrm{b}}_{\mathrm{}}^{\mathrm{}}$-tagged AK4 jet not overlapping with $j_{{{\mathrm{H}}_{\mathrm{}}^{\mathrm{}}}}$ such that $\varDelta R(j,j_{{{\mathrm{H}}_{\mathrm{}}^{\mathrm{}}}})>1.2$ is satisfied. Since the resonance mass cannot be reconstructed because of the presence of undetected decay products, the $j_{{{\mathrm{H}}_{\mathrm{}}^{\mathrm{}}}}$ momentum and the ${\vec p}_{\mathrm {T}}^{\text {miss}}$ are used to compute the transverse mass $m_{{{\mathrm{X}}_{\mathrm{}}^{\mathrm{}}}}^{\text {T}} = \sqrt{\smash [b]{2p_{\mathrm {T}} ^\text {miss} p_{\mathrm {T}} ^{{\mathrm{H}}_{\mathrm{}}^{\mathrm{}}} (1-\cos \varDelta \phi ({\vec p}_{\mathrm {T}}^{\text {miss}},{\vec p}_{\mathrm {T}} ^{{\mathrm{H}}_{\mathrm{}}^{\mathrm{}}}))}}$. In the VBF category, the condition $|\eta _{j_{{\mathrm{H}}_{\mathrm{}}^{\mathrm{}}}} |<1.1$ is applied on the $j_{{{\mathrm{H}}_{\mathrm{}}^{\mathrm{}}}}$ to reduce the contribution of events where the measured $m_{{{\mathrm{X}}_{\mathrm{}}^{\mathrm{}}}}^{\text {T}}$ is significantly below $m_{{{\mathrm{{{\mathrm{Z}}_{\mathrm{}}^{\mathrm{}}}}}_{\mathrm{}}^{\mathrm{\prime }}}}$.

For the 2${\mathrm{e}}_{\mathrm{}}^{\mathrm{}}$ categories, data are collected using an electron trigger that requires either an isolated electron with $p_{\mathrm {T}} > 35\,\text {Ge}\text {V}$ or a nonisolated electron with $p_{\mathrm {T}} > 115\,\text {Ge}\text {V}$. In the 2$\upmu$ categories, a muon trigger that requires a nonisolated muon with $p_{\mathrm {T}} > 50\,\text {Ge}\text {V}$ is used to collect data. For both the 2${\mathrm{e}}_{\mathrm{}}^{\mathrm{}}$ and 2$\upmu$ categories, the two selected leptons must have opposite charge, $p_{\mathrm {T}} > 55$ and 20$\,\text {Ge}\text {V}$, respectively, and should be isolated from other activity in the event, except for each other. The ${\mathrm{Z}}_{\mathrm{}}^{\mathrm{}}$ boson candidates are required to have a dilepton invariant mass in the range 70–110$\,\text {Ge}\text {V}$, and $p_{\mathrm {T}} > 200\,\text {Ge}\text {V}$. The ${\mathrm{Z}}_{\mathrm{}}^{\mathrm{}}$ boson mass window is large compared with the dilepton mass resolution, which is 3 (4)% for an electron (muon) pair. A more stringent selection would decrease both the signal and the ${{\mathrm{Z}}_{\mathrm{}}^{\mathrm{}}} \text {+jets}$ background selection efficiency by the same amount, thus reducing the signal sensitivity. The separation between the ${\mathrm{Z}}_{\mathrm{}}^{\mathrm{}}$ boson candidate and $j_{{{\mathrm{H}}_{\mathrm{}}^{\mathrm{}}}}$ is required to be $\varDelta R(j_{{{\mathrm{H}}_{\mathrm{}}^{\mathrm{}}}},Z) > 2$ for all categories, and $|\varDelta \eta (j_{{{\mathrm{H}}_{\mathrm{}}^{\mathrm{}}}},Z) | < 1.7$ additionally for the non-VBF categories, to further reduce the ${{\mathrm{Z}}_{\mathrm{}}^{\mathrm{}}} \text {+jets}$ background.

Candidate VBF events are selected in both the $0\ell$ and $2\ell$ categories by requiring two additional AK4 jets (j) with $|\eta _j |<5$ that satisfy $\varDelta R(j,j_{{{\mathrm{H}}_{\mathrm{}}^{\mathrm{}}}})>1.2$ in order to avoid overlap with the $j_{{{\mathrm{H}}_{\mathrm{}}^{\mathrm{}}}}$, have $\eta _j$ values of opposite sign, a dijet mass $m_{jj} > 500\,\text {Ge}\text {V}$, and that satisfy a separation $\varDelta \eta _{jj} > 4$. The two AK4 jets with the highest dijet mass are selected.

**Fig. 2:**
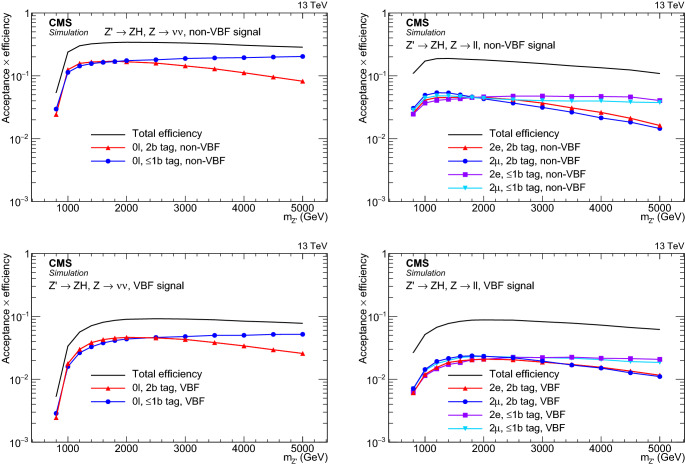
The product of signal acceptance and efficiency in the $0\ell$ (left column) and $2\ell$ (right column) categories for the signal produced via ${{\mathrm{q}}_{\mathrm{}}^{\mathrm{}}} {{\overline{{{{\mathrm{q}}_{\mathrm{}}^{\mathrm{}}}}}}}$ annihilation (upper row) and vector boson fusion (lower row)

A further requirement is to have either $m_{{{\mathrm{X}}_{\mathrm{}}^{\mathrm{}}}}$ or $m_{{{\mathrm{X}}_{\mathrm{}}^{\mathrm{}}}}^{\text {T}}$ larger than 1200$\,\text {Ge}\text {V}$ for the $\le$1${\mathrm{b}}_{\mathrm{}}^{\mathrm{}}$ tag, non-VBF categories, and larger than 750$\,\text {Ge}\text {V}$ for the other categories to ensure the smoothness of the background model. The product of the signal geometrical acceptance and the selection efficiency, reported in Fig. 2, is calculated for the $0\ell$ category with the denominator being the ${\mathrm{Z}}_{\mathrm{}}^{\mathrm{}}$ decay to neutrinos, and for the $2\ell$ categories with the denominator being the ${\mathrm{Z}}_{\mathrm{}}^{\mathrm{}}$ decay to electrons, muons and tau leptons.

## Background estimation and signal modeling

The most important SM background is vector boson production in association with ${\mathrm{b}}_{\mathrm{}}^{\mathrm{}}$-tagged jets (${{\mathrm{V}}_{\mathrm{}}^{\mathrm{}}} \text {+jets}$). The ${{\mathrm{V}}_{\mathrm{}}^{\mathrm{}}} \text {+jets}$ background is estimated using control samples in data to reduce the dependence on simulation. Minor SM backgrounds are ${\mathrm{t}}_{\mathrm{}}^{\mathrm{}}$ $\overline{{{{\mathrm{t}}_{\mathrm{}}^{\mathrm{}}}}}$ and single top quark processes, SM diboson production (${{\mathrm{V}}_{\mathrm{}}^{\mathrm{}}} {{\mathrm{V}}_{\mathrm{}}^{\mathrm{}}}$), and SM ${\mathrm{H}}_{\mathrm{}}^{\mathrm{}}$ production in association with a vector boson (${{\mathrm{V}}_{\mathrm{}}^{\mathrm{}}} {{\mathrm{H}}_{\mathrm{}}^{\mathrm{}}}$), all of which are estimated based on simulation. The SM ${{\mathrm{Z}}_{\mathrm{}}^{\mathrm{}}} {{\mathrm{H}}_{\mathrm{}}^{\mathrm{}}}$ production is considered as a background in this analysis. However, this process can be distinguished from the signal because of the non-resonant distribution in the ${{\mathrm{Z}}_{\mathrm{}}^{\mathrm{}}} {{\mathrm{H}}_{\mathrm{}}^{\mathrm{}}}$ invariant mass and by the softer $p_{\mathrm {T}}$ spectra of the ${\mathrm{H}}_{\mathrm{}}^{\mathrm{}}$ and ${\mathrm{Z}}_{\mathrm{}}^{\mathrm{}}$ bosons. The jet mass distribution is split into a signal-enriched region (SR) with $105<m_{j_{{{\mathrm{H}}_{\mathrm{}}^{\mathrm{}}}}} <135\,\text {Ge}\text {V}$, and low-mass and high-mass sidebands (SB) with $30<m_{j_{{{\mathrm{H}}_{\mathrm{}}^{\mathrm{}}}}} <65\,\text {Ge}\text {V}$ (LSB) and $135<m_{j_{{{\mathrm{H}}_{\mathrm{}}^{\mathrm{}}}}} <250\,\text {Ge}\text {V}$ (HSB), respectively. The jet mass range $65<m_{j_{{{\mathrm{H}}_{\mathrm{}}^{\mathrm{}}}}} <105\,\text {Ge}\text {V}$, a region enriched with boosted vector bosons (VR), is excluded and kept blinded in order to avoid potential contamination from a ${{\mathrm{V}}_{\mathrm{}}^{\mathrm{}}} {{\mathrm{V}}_{\mathrm{}}^{\mathrm{}}}$ resonant signal, which is the subject of dedicated searches [16, 62, 63]. The background estimation consists of two separate steps to determine, first, the number of events and, second, the distribution of the main background in the SR.

**Table 2:** Scale factors derived for the normalization of the ${\mathrm{t}}_{\mathrm{}}^{\mathrm{}}$ $\overline{{{{\mathrm{t}}_{\mathrm{}}^{\mathrm{}}}}}$ and single top quark backgrounds for different event categories. Uncertainties due to the limited size of the event samples (stat.) and systematic effects (syst.) are reported as well. The scale factors of the 2${\mathrm{e}}_{\mathrm{}}^{\mathrm{}}$ and 2$\upmu$ categories are derived using the $1{{\mathrm{e}}_{\mathrm{}}^{\mathrm{}}} 1{\upmu {}{}}$ top quark control region as described in the text

Non-VBF category		${\mathrm{t}}_{\mathrm{}}^{\mathrm{}}$ $\overline{{{{\mathrm{t}}_{\mathrm{}}^{\mathrm{}}}}}$, single top quark SF ± stat. ± syst.
2${\mathrm{b}}_{\mathrm{}}^{\mathrm{}}$ tag	$0\ell$	$1.012\pm 0.116 \pm 0.008$
	$2{{\mathrm{e}}_{\mathrm{}}^{\mathrm{}}}$	$1.098\pm 0.084 \pm 0.067$
	$2{\upmu {}{}}$	$1.098\pm 0.084 \pm 0.075$
$\le$1${\mathrm{b}}_{\mathrm{}}^{\mathrm{}}$ tag	$0\ell$	$1.028\pm 0.048 \pm 0.009$
	$2{{\mathrm{e}}_{\mathrm{}}^{\mathrm{}}}$	$1.003\pm 0.021 \pm 0.089$
	$2{\upmu {}{}}$	$1.003\pm 0.021 \pm 0.095$

VBF category		${\mathrm{t}}_{\mathrm{}}^{\mathrm{}}$ $\overline{{{{\mathrm{t}}_{\mathrm{}}^{\mathrm{}}}}}$, single top quark SF ± stat. ± syst. ± VBF norm.
2${\mathrm{b}}_{\mathrm{}}^{\mathrm{}}$ tag	$0\ell$	$0.676\pm 0.221 \pm 0.007\pm 0.330$
	$2{{\mathrm{e}}_{\mathrm{}}^{\mathrm{}}}$	$0.676\pm 0.154 \pm 0.096\pm 0.330$
	$2{\upmu {}{}}$	$0.676\pm 0.154 \pm 0.103\pm 0.330$
$\le$1${\mathrm{b}}_{\mathrm{}}^{\mathrm{}}$ tag	$0\ell$	$0.822\pm 0.144 \pm 0.022\pm 0.180$
	$2{{\mathrm{e}}_{\mathrm{}}^{\mathrm{}}}$	$0.882\pm 0.044 \pm 0.099\pm 0.120$
	$2{\upmu {}{}}$	$0.882\pm 0.044 \pm 0.107\pm 0.120$

### Background normalization

The three groups of backgrounds (${{\mathrm{V}}_{\mathrm{}}^{\mathrm{}}} \text {+jets}$, ${\mathrm{t}}_{\mathrm{}}^{\mathrm{}}$ $\overline{{{{\mathrm{t}}_{\mathrm{}}^{\mathrm{}}}}}$ and single top quark, and ${{\mathrm{V}}_{\mathrm{}}^{\mathrm{}}} {{\mathrm{V}}_{\mathrm{}}^{\mathrm{}}}$ and ${{\mathrm{V}}_{\mathrm{}}^{\mathrm{}}} {{\mathrm{H}}_{\mathrm{}}^{\mathrm{}}}$) are considered separately, since each group has different physical properties leading to a different shape of the jet mass distribution. An appropriate analytical function is chosen to describe the background in each case. The ${{\mathrm{V}}_{\mathrm{}}^{\mathrm{}}} \text {+jets}$ background’s Higgs candidate jet mass has a smoothly falling shape with no peaks, therefore Chebyshev polynomials of order 1–4 are chosen to model the distribution observed in data. The ${{\mathrm{V}}_{\mathrm{}}^{\mathrm{}}} {{\mathrm{V}}_{\mathrm{}}^{\mathrm{}}}$ and ${{\mathrm{V}}_{\mathrm{}}^{\mathrm{}}} {{\mathrm{H}}_{\mathrm{}}^{\mathrm{}}}$ backgrounds have two peaks in the jet mass distribution, corresponding to the ${\mathrm{W}}_{\mathrm{}}^{\mathrm{}}$ and ${\mathrm{Z}}_{\mathrm{}}^{\mathrm{}}$ bosons, and the ${{\mathrm{V}}_{\mathrm{}}^{\mathrm{}}} {{\mathrm{H}}_{\mathrm{}}^{\mathrm{}}}$ background an additional peak due to the Higgs boson. The ${\mathrm{t}}_{\mathrm{}}^{\mathrm{}}$ $\overline{{{{\mathrm{t}}_{\mathrm{}}^{\mathrm{}}}}}$ and single top quark backgrounds are considered together, because they both have two peaks corresponding to ${{\mathrm{W}}_{\mathrm{}}^{\mathrm{}}} \rightarrow {{\mathrm{q}}_{\mathrm{}}^{\mathrm{}}} {{\overline{{{{\mathrm{q}}_{\mathrm{}}^{\mathrm{}}}}}}} '$ decays and all-hadronic top quark decays ${{\mathrm{t}}_{\mathrm{}}^{\mathrm{}}}\rightarrow {{\mathrm{W}}_{\mathrm{}}^{\mathrm{}}} {{\mathrm{b}}_{\mathrm{}}^{\mathrm{}}} \rightarrow {{\mathrm{q}}_{\mathrm{}}^{\mathrm{}}} {{\overline{{{{\mathrm{q}}_{\mathrm{}}^{\mathrm{}}}}}}} '{{\mathrm{b}}_{\mathrm{}}^{\mathrm{}}}$.

**Fig. 3:**
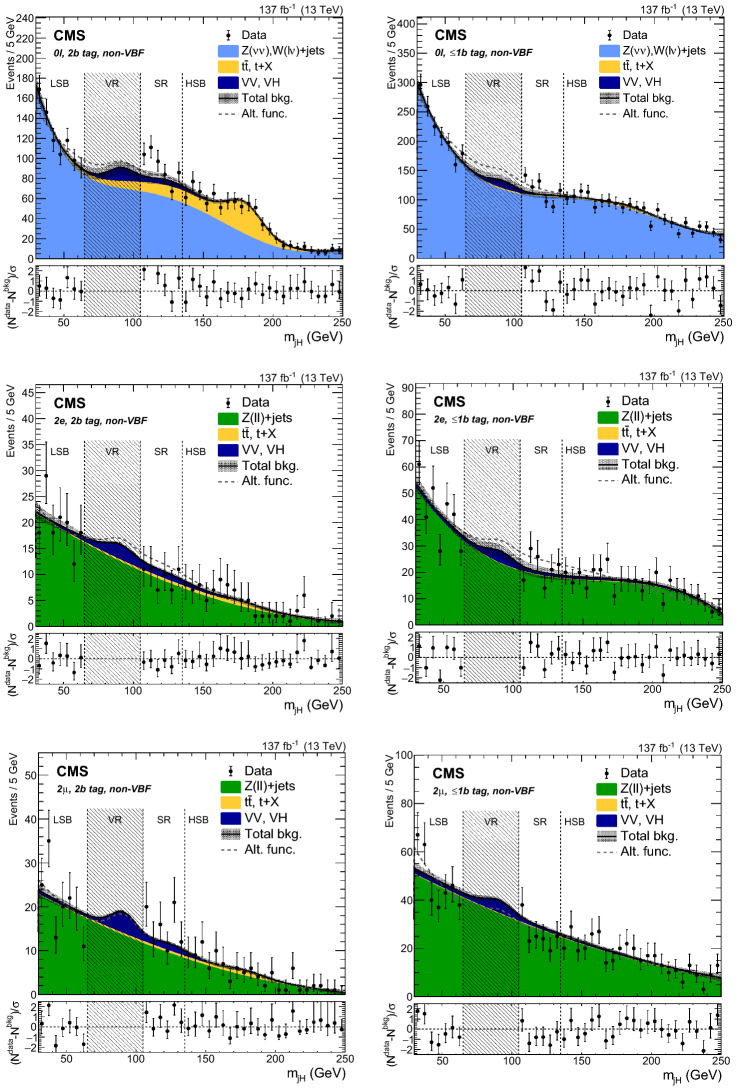
Fit to the $m_{j_{{{\mathrm{H}}_{\mathrm{}}^{\mathrm{}}}}}$ distribution in data in the 2${\mathrm{b}}_{\mathrm{}}^{\mathrm{}}$ tag (left column) and $\le$1${\mathrm{b}}_{\mathrm{}}^{\mathrm{}}$ tag (right column) non-VBF categories, for $0\ell$ (upper row), 2${\mathrm{e}}_{\mathrm{}}^{\mathrm{}}$ (middle row), and 2$\upmu$ (lower row). The shaded bands around the total background estimate represent the uncertainty from the fit to data in the jet mass SBs. The observed data are indicated by black markers. The vertical shaded band indicates the VR region, which is blinded and not used in the fit to avoid potential contamination from ${{\mathrm{V}}_{\mathrm{}}^{\mathrm{}}} {{\mathrm{V}}_{\mathrm{}}^{\mathrm{}}}$ resonant signals. The dashed vertical lines separate the LSB, VR, SR, and HSB. The bottom panel shows $(N^{\text {data}}-N^{\text {bkg}})/\sigma$ for each bin, where $\sigma$ is the statistical uncertainty in data. In the $\le$1${\mathrm{b}}_{\mathrm{}}^{\mathrm{}}$ tag, non-VBF categories, $m_{{{\mathrm{X}}_{\mathrm{}}^{\mathrm{}}}}$ or $m_{{{\mathrm{X}}_{\mathrm{}}^{\mathrm{}}}}^{\text {T}}$ are required to be larger than 1200$\,\text {Ge}\text {V}$ to ensure the smoothness of the background model

**Fig. 4:**
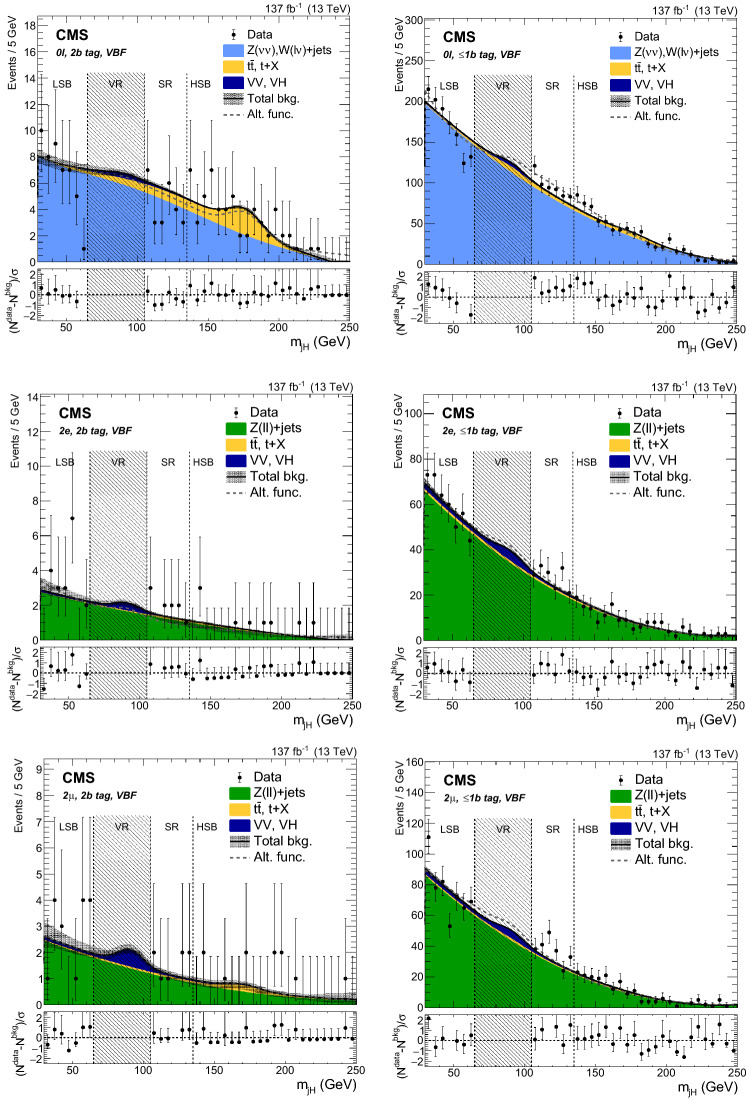
Fit to the $m_{j_{{{\mathrm{H}}_{\mathrm{}}^{\mathrm{}}}}}$ distribution in data in the 2${\mathrm{b}}_{\mathrm{}}^{\mathrm{}}$ tag (left column) and $\le$1${\mathrm{b}}_{\mathrm{}}^{\mathrm{}}$ tag (right column) VBF categories, for $0\ell$ (upper row), 2${\mathrm{e}}_{\mathrm{}}^{\mathrm{}}$ (middle row), and 2$\upmu$ (lower row). The shaded bands around the total background estimate represent the uncertainty from the fit to data in the jet mass SBs. The observed data are indicated by black markers. The observed data are indicated by black markers. The vertical shaded band indicates the VR region, which is blinded and not used in the fit to avoid potential contamination from ${{\mathrm{V}}_{\mathrm{}}^{\mathrm{}}} {{\mathrm{V}}_{\mathrm{}}^{\mathrm{}}}$ resonant signals. The dashed vertical lines separate the LSB, VR, SR, and HSB. The bottom panel shows $(N^{\text {data}}-N^{\text {bkg}})/\sigma$ for each bin, where $\sigma$ is the statistical uncertainty in data

The normalization of the simulated top quark background is corrected with a scale factor (SF) determined in high-purity top quark control regions. In the $0\ell$ category, the control region is defined by the veto on the additional ${\mathrm{b}}_{\mathrm{}}^{\mathrm{}}$-tagged AK4 jet being inverted. In the $2\ell$ categories, control region data are collected using the same trigger as for the 2${\mathrm{e}}_{\mathrm{}}^{\mathrm{}}$ signal region, with a requirement that lepton flavors and charges are different, resulting in a $1{{\mathrm{e}}_{\mathrm{}}^{\mathrm{}}} 1{\upmu {}{}}$ region, where the leptons must have a combined invariant mass $m_{{{\mathrm{e}}_{\mathrm{}}^{\mathrm{}}} {\upmu {}{}}} > 110$ $\,\text {Ge}\text {V}$ and a vector sum $p_{\mathrm {T}} ^{{{\mathrm{e}}_{\mathrm{}}^{\mathrm{}}} {\upmu {}{}}} > 120$ $\,\text {Ge}\text {V}$. Multiplicative SFs are calculated from the ratio of the event yield between data and simulation and are applied to the simulated samples in the SR. The uncertainties in the top quark SFs originate from the limited event count in the top quark control region and the extrapolation from the top quark control region to the SR. The systematic uncertainty in the $0\ell$ category is derived by varying the ${\mathrm{b}}_{\mathrm{}}^{\mathrm{}}$ tagging SF. For the $2\ell$ categories the uncertainties in the electron and muon identification are taken into account. The electron and muon trigger uncertainties only affect the 2$\upmu$ and not the 2${\mathrm{e}}_{\mathrm{}}^{\mathrm{}}$ category because the electron trigger is used to provide the control region while the muon trigger is used to select the signal region. A normalization uncertainty is applied to the VBF categories to account for the limited event counts in these control regions. The normalization uncertainty is taken as the deviation of the top quark SF from unity as shown in Table 2.

The background model, composed of the sum of the ${{\mathrm{V}}_{\mathrm{}}^{\mathrm{}}} \text {+jets}$, ${\mathrm{t}}_{\mathrm{}}^{\mathrm{}}$ $\overline{{{{\mathrm{t}}_{\mathrm{}}^{\mathrm{}}}}}$ and single top quark, and the ${{\mathrm{V}}_{\mathrm{}}^{\mathrm{}}} {{\mathrm{V}}_{\mathrm{}}^{\mathrm{}}}$ and ${{\mathrm{V}}_{\mathrm{}}^{\mathrm{}}} {{\mathrm{H}}_{\mathrm{}}^{\mathrm{}}}$ templates is fitted to the SBs of the jet mass distribution in data. The analytical function parameters and the normalization of the top quark and ${{\mathrm{V}}_{\mathrm{}}^{\mathrm{}}} {{\mathrm{V}}_{\mathrm{}}^{\mathrm{}}}$ backgrounds are fixed from the fit to simulation, but the shape parameters from the ${{\mathrm{V}}_{\mathrm{}}^{\mathrm{}}} \text {+jets}$ background are not. The number of parameters for the fit to data is determined by a Fisher F-test [64]. The number of expected events is derived from the integral of the fitted model in the SR. The choice of the ${{\mathrm{V}}_{\mathrm{}}^{\mathrm{}}} \text {+jets}$ fit function induces a systematic uncertainty, which can be determined by fitting the ${{\mathrm{V}}_{\mathrm{}}^{\mathrm{}}} \text {+jets}$ background shape with an alternative function, consisting of the sum of an exponential and a Gaussian function, and considering the difference between the integrals of the two fit models in the SR as a systematic uncertainty. Figures 3 and 4 show the fits to the jet mass in the different categories. Table 3 summarizes the expected background yield in the SR.

**Table 3:** The expected and observed numbers of background events in the signal region for all event categories. The ${{\mathrm{V}}_{\mathrm{}}^{\mathrm{}}} \text {+jets}$ background uncertainties originate from the variation of the parameters within the fit uncertainties (fit) and the difference between the nominal and alternative function choice for the fit to $m_{j_{{{\mathrm{H}}_{\mathrm{}}^{\mathrm{}}}}}$ (alt). The ${\mathrm{t}}_{\mathrm{}}^{\mathrm{}}$ $\overline{{{{\mathrm{t}}_{\mathrm{}}^{\mathrm{}}}}}$ and single top quark uncertainties arise from the $m_{j_{{{\mathrm{H}}_{\mathrm{}}^{\mathrm{}}}}}$ modeling, the statistical component of the top quark SF uncertainties, and the extrapolation uncertainty from the control region to the SR. The ${{\mathrm{V}}_{\mathrm{}}^{\mathrm{}}} {{\mathrm{V}}_{\mathrm{}}^{\mathrm{}}}$ and ${{\mathrm{V}}_{\mathrm{}}^{\mathrm{}}} {{\mathrm{H}}_{\mathrm{}}^{\mathrm{}}}$ normalization uncertainties come from the $m_{j_{{{\mathrm{H}}_{\mathrm{}}^{\mathrm{}}}}}$ modeling

Non-VBF category		${{\mathrm{V}}_{\mathrm{}}^{\mathrm{}}} \text {+jets}$ (±fit) (±alt)	${\mathrm{t}}_{\mathrm{}}^{\mathrm{}}$ $\overline{{{{\mathrm{t}}_{\mathrm{}}^{\mathrm{}}}}}$, single top quark	${{\mathrm{V}}_{\mathrm{}}^{\mathrm{}}} {{\mathrm{V}}_{\mathrm{}}^{\mathrm{}}}$, ${{\mathrm{V}}_{\mathrm{}}^{\mathrm{}}} {{\mathrm{H}}_{\mathrm{}}^{\mathrm{}}}$	Bkg. sum	Observed
2${\mathrm{b}}_{\mathrm{}}^{\mathrm{}}$ tag	$0\ell$	$374 \pm 34 \pm 20$	$68\pm 8$	$31\pm 10$	$474\pm 42$	549
	$2{{\mathrm{e}}_{\mathrm{}}^{\mathrm{}}}$	$54 \pm 5 \pm 8$	$3.1\pm 0.4$	$7.9\pm 1.9$	$65\pm 10$	57
	$2{\upmu {}{}}$	$60 \pm 5 \pm 1$	$3.2\pm 0.6$	$9.1\pm 2.1$	$72\pm 5$	91
$\le$1${\mathrm{b}}_{\mathrm{}}^{\mathrm{}}$ tag	$0\ell$	$637 \pm 35 \pm 51$	$7.3\pm 0.9$	$15\pm 4$	$659\pm 61$	697
	$2{{\mathrm{e}}_{\mathrm{}}^{\mathrm{}}}$	$113 \pm 14 \pm 27$	$1.6\pm 0.2$	$7.2\pm 1.7$	$122\pm 31$	130
	$2{\upmu {}{}}$	$167 \pm 8 \pm 10$	$1.8\pm 0.2$	$8.0\pm 1.8$	$177\pm 13$	154

VBF category		${{\mathrm{V}}_{\mathrm{}}^{\mathrm{}}} \text {+jets}$ (±fit) (±alt)	${\mathrm{t}}_{\mathrm{}}^{\mathrm{}}$ $\overline{{{{\mathrm{t}}_{\mathrm{}}^{\mathrm{}}}}}$, single top quark	${{\mathrm{V}}_{\mathrm{}}^{\mathrm{}}} {{\mathrm{V}}_{\mathrm{}}^{\mathrm{}}}$, ${{\mathrm{V}}_{\mathrm{}}^{\mathrm{}}} {{\mathrm{H}}_{\mathrm{}}^{\mathrm{}}}$	Bkg. sum	Observed
2${\mathrm{b}}_{\mathrm{}}^{\mathrm{}}$ tag	$0\ell$	$28 \pm 3 \pm 3$	$4.3\pm 2.0$	$0.9\pm 0.6$	$33\pm 5$	26
	$2{{\mathrm{e}}_{\mathrm{}}^{\mathrm{}}}$	$7.3 \pm 2.0 \pm 2.0$	$0.4\pm 0.2$	$0.4\pm 0.1$	$8.1\pm 2.8$	10
	$2{\upmu {}{}}$	$6.0 \pm 1.7 \pm 0.2$	$0.4\pm 0.2$	$0.5\pm 0.1$	$7.0\pm 1.7$	8.0
$\le$1${\mathrm{b}}_{\mathrm{}}^{\mathrm{}}$ tag	$0\ell$	$486 \pm 13 \pm 72$	$25\pm 6$	$6.3\pm 1.5$	$517\pm 73$	572
	$2{{\mathrm{e}}_{\mathrm{}}^{\mathrm{}}}$	$137 \pm 7 \pm 7$	$4.8\pm 1.5$	$6.4\pm 1.5$	$148\pm 10$	168
	$2{\upmu {}{}}$	$171 \pm 8 \pm 6$	$4.5\pm 1.1$	$7.7\pm 1.8$	$183\pm 10$	222

**Fig. 5:**
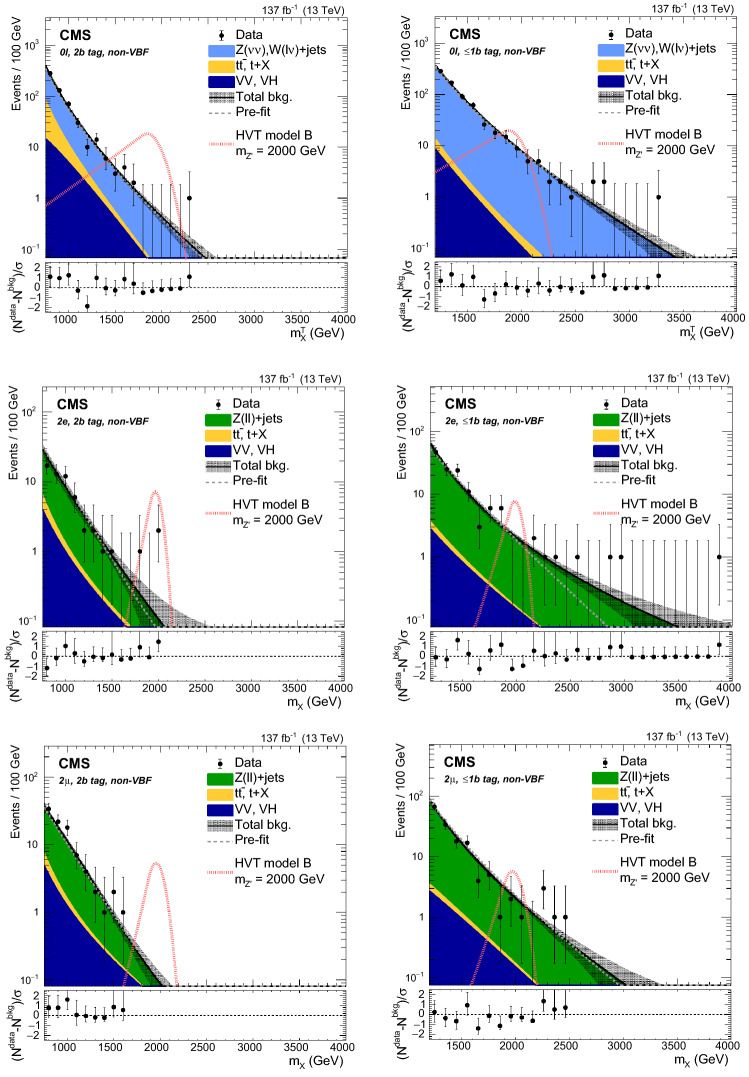
Distributions in data in the 2${\mathrm{b}}_{\mathrm{}}^{\mathrm{}}$ tag (left column) and $\le$1${\mathrm{b}}_{\mathrm{}}^{\mathrm{}}$ tag (right column) non-VBF categories, of $m_{{{\mathrm{X}}_{\mathrm{}}^{\mathrm{}}}}^{\text {T}}$ for $0\ell$ (upper row), and $m_{{{\mathrm{X}}_{\mathrm{}}^{\mathrm{}}}}$ for 2${\mathrm{e}}_{\mathrm{}}^{\mathrm{}}$ (middle row), and 2$\upmu$ (lower row). The distributions are shown up to 4000$\,\text {Ge}\text {V}$, which corresponds to the event with the highest $m_{{{\mathrm{X}}_{\mathrm{}}^{\mathrm{}}}}$ or $m_{{{\mathrm{X}}_{\mathrm{}}^{\mathrm{}}}}^{\text {T}}$ observed in the SR. The shaded bands represent the uncertainty from the background estimation. The observed data are represented by black markers, and the potential contribution of a resonance produced in the context of the HVT model B at $m_{{{\mathrm{{{\mathrm{Z}}_{\mathrm{}}^{\mathrm{}}}}}_{\mathrm{}}^{\mathrm{\prime }}}} =2000\,\text {Ge}\text {V}$ is shown as a dotted red line. The bottom panel shows $(N^{\text {data}}-N^{\text {bkg}})/\sigma$ for each bin, where $\sigma$ is the statistical uncertainty in data

### Background distribution

The $m_{{{\mathrm{X}}_{\mathrm{}}^{\mathrm{}}}}$ and $m_{{{\mathrm{X}}_{\mathrm{}}^{\mathrm{}}}}^{\text {T}}$ distributions are estimated using the data in the jet mass SBs. An $\alpha$ function is then defined as the ratio of the two functions describing the simulated $m_{{{\mathrm{X}}_{\mathrm{}}^{\mathrm{}}}}$ (or $m_{{{\mathrm{X}}_{\mathrm{}}^{\mathrm{}}}}^{\text {T}}$) shape in the SR and SB region of the ${{\mathrm{V}}_{\mathrm{}}^{\mathrm{}}} \text {+jets}$ background:

1 $$\begin{aligned} \alpha (m) = \frac{N_{\text {SR}}^{{{\mathrm{V}}_{\mathrm{}}^{\mathrm{}}} \text {+jets}}(m)}{N_{\text {SB}}^{{{\mathrm{V}}_{\mathrm{}}^{\mathrm{}}} \text {+jets}}(m)}, \end{aligned}$$

where *N* denotes the function and *m* represents either $m_{{{\mathrm{X}}_{\mathrm{}}^{\mathrm{}}}}$ or $m_{{{\mathrm{X}}_{\mathrm{}}^{\mathrm{}}}}^{\text {T}}$. The functions are normalized to the number of events derived in Sect. 6.1 and shown in Table 3.

The ${{\mathrm{V}}_{\mathrm{}}^{\mathrm{}}} \text {+jets}$ background shape in the SR is thus estimated as the product of $\alpha (m)$ and the shape in the data SBs after subtracting the corresponding top quark and ${{\mathrm{V}}_{\mathrm{}}^{\mathrm{}}} {{\mathrm{V}}_{\mathrm{}}^{\mathrm{}}}$ contributions:

2 $$\begin{aligned} N_{\text {SR}}^{{{\mathrm{V}}_{\mathrm{}}^{\mathrm{}}} \text {+jets}}(m) = \left[ N_{\text {SB}}^{\text {data}}(m) - N_{\text {SB}}^{\text {top}}(m) - N_{\text {SB}}^{\text {VV}}(m) \right] \alpha (m). \end{aligned}$$

Finally, the expected number of background events in the SR is derived by adding the top quark and ${{\mathrm{V}}_{\mathrm{}}^{\mathrm{}}} {{\mathrm{V}}_{\mathrm{}}^{\mathrm{}}}$ contributions to the ${{\mathrm{V}}_{\mathrm{}}^{\mathrm{}}} \text {+jets}$ background distribution and taking the ${{\mathrm{V}}_{\mathrm{}}^{\mathrm{}}} \text {+jets}$ normalization from the fit to data in the jet mass SBs:

3 $$\begin{aligned} N_{\text {SR}}^{\text {bkg}}(m) = N_{\text {SR}}^{{{\mathrm{V}}_{\mathrm{}}^{\mathrm{}}} \text {+jets}}(m) + N_{\text {SR}}^{\text {top}}(m) + N_{\text {SR}}^{\text {VV}}(m). \end{aligned}$$

**Fig. 6:**
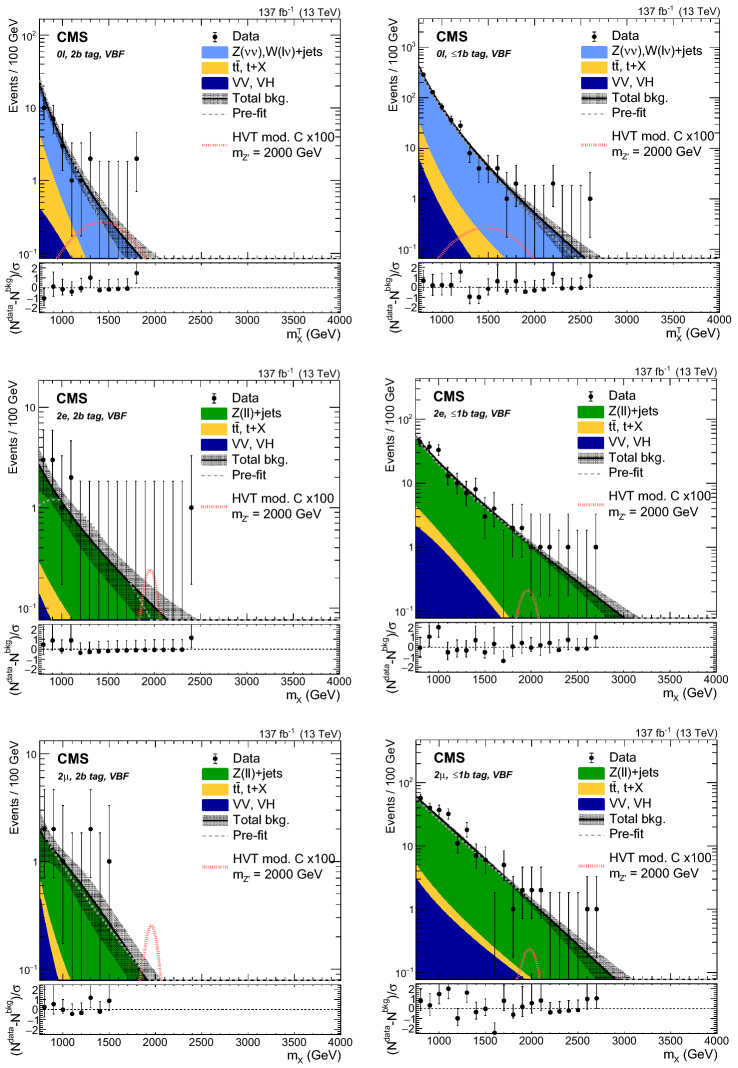
Distributions in data in the 2${\mathrm{b}}_{\mathrm{}}^{\mathrm{}}$ tag (left column) and $\le$1${\mathrm{b}}_{\mathrm{}}^{\mathrm{}}$ tag (right column) VBF categories, of $m_{{{\mathrm{X}}_{\mathrm{}}^{\mathrm{}}}}^{\text {T}}$ for $0\ell$ (upper row), and $m_{{{\mathrm{X}}_{\mathrm{}}^{\mathrm{}}}}$ for 2${\mathrm{e}}_{\mathrm{}}^{\mathrm{}}$ (middle row), and 2$\upmu$ (lower row). The distributions are shown up to 4000$\,\text {Ge}\text {V}$, which corresponds to the event with the highest $m_{{{\mathrm{X}}_{\mathrm{}}^{\mathrm{}}}}$ or $m_{{{\mathrm{X}}_{\mathrm{}}^{\mathrm{}}}}^{\text {T}}$ observed in the SR. The shaded bands represent the uncertainty from the background estimation. The observed data are represented by black markers, and the potential contribution of a resonance produced in the context of the HVT model C at $m_{{{\mathrm{{{\mathrm{Z}}_{\mathrm{}}^{\mathrm{}}}}}_{\mathrm{}}^{\mathrm{\prime }}}} =2000\,\text {Ge}\text {V}$ is shown as a dotted red line. The bottom panel shows $(N^{\text {data}}-N^{\text {bkg}})/\sigma$ for each bin, where $\sigma$ is the statistical uncertainty in data

The observed data, along with the expected backgrounds, are reported for each category in Figs. 5 and 6.

The background estimation method is validated by splitting the LSB in two regions: $30<m_{j_{{{\mathrm{H}}_{\mathrm{}}^{\mathrm{}}}}} <50\,\text {Ge}\text {V}$ and $50<m_{j_{{{\mathrm{H}}_{\mathrm{}}^{\mathrm{}}}}} <65\,\text {Ge}\text {V}$. The first one is used as a new LSB and the second one as a proxy for the SR. The data yields and distributions are found to be compatible with the expectation in all categories.

### Signal modeling

In order to build a template for the signal extraction, the simulated signal mass points are fitted in the SR with the Crystal Ball function [65], which consists of a Gaussian core and a power-law function that describes the low-end tail below a certain threshold. The parameterization for intermediate mass points is determined by linearly interpolating the shape parameters derived by fitting the generated mass points.

## Systematic uncertainties

The systematic uncertainty in the ${{\mathrm{V}}_{\mathrm{}}^{\mathrm{}}} \text {+jets}$ background is dominated by the statistical uncertainty of the number of data events in the SBs. The systematic uncertainties in the shape of the ${{\mathrm{V}}_{\mathrm{}}^{\mathrm{}}} \text {+jets}$ background are estimated from the covariance matrix of the simultaneous fit of the $m_{{{\mathrm{X}}_{\mathrm{}}^{\mathrm{}}}}^{\text {T}}$ and $m_{{{\mathrm{X}}_{\mathrm{}}^{\mathrm{}}}}$ distributions in data in the SBs, and in simulated ${{\mathrm{V}}_{\mathrm{}}^{\mathrm{}}} \text {+jets}$ background events in the signal and SB regions. Most of the effect of the uncertainties is correlated among the SB and SR, and cancels out in the $\alpha$ ratio. The ${\mathrm{t}}_{\mathrm{}}^{\mathrm{}}$ $\overline{{{{\mathrm{t}}_{\mathrm{}}^{\mathrm{}}}}}$ and ${{\mathrm{V}}_{\mathrm{}}^{\mathrm{}}} {{\mathrm{V}}_{\mathrm{}}^{\mathrm{}}}$ background shape uncertainties are propagated from the covariance matrix of the fit to the simulation in the SR. The statistical treatment is consistent with Ref. [16].

The uncertainty in the top quark background normalization originates from a limited event count in data and simulated event samples in the control regions, and from the variations on the requirements of lepton selection, ${\mathrm{b}}_{\mathrm{}}^{\mathrm{}}$ tagging SFs, and the VBF selection used to select events in the control region. The uncertainties are reported in Table 2. The uncertainties in the trigger, identification, and isolation efficiencies of leptons affect the normalization and shape of the simulated signal and diboson background. The uncertainties are evaluated by moving the SFs, derived as the efficiency in data over the efficiency in simulation, up and down by one standard deviation, and amount to 1–7%.

The lepton scale and resolution affect both shape and normalization of the signal, leading to an uncertainty of 1–3%. The uncertainty from the effect of the $p_{\mathrm {T}} ^\text {miss}$ scale and resolution on the normalization of the signal and ${{\mathrm{V}}_{\mathrm{}}^{\mathrm{}}} {{\mathrm{V}}_{\mathrm{}}^{\mathrm{}}}$,${{\mathrm{V}}_{\mathrm{}}^{\mathrm{}}} {{\mathrm{H}}_{\mathrm{}}^{\mathrm{}}}$ background is 1%. The jet energy scale and resolution uncertainties amount to a 1% systematic uncertainty in the normalization and a shape variation in the distribution of the signal and diboson background events. The uncertainty in the jet mass scale (resolution) adds a contribution of 0.6 (9.0)%) to the uncertainty in the signal and the diboson background normalization. The jet mass scale and resolution depend on the choice of the parton shower model, which affects the Higgs boson tagging and leads to an additional uncertainty of 6% in the signal normalization. The uncertainty was evaluated by using herwig++ 2.7.1 [66] as an alternative showering algorithm. The impact of the ${\mathrm{b}}_{\mathrm{}}^{\mathrm{}}$ tagging systematic uncertainty in the signal efficiency depends on the mass of the resonance and has a range of 4–15% for the 2${\mathrm{b}}_{\mathrm{}}^{\mathrm{}}$ tag categories and 1–6% for the $\le$1${\mathrm{b}}_{\mathrm{}}^{\mathrm{}}$ tag categories. The uncertainty is treated as anti-correlated between the two ${\mathrm{b}}_{\mathrm{}}^{\mathrm{}}$ tag categories.

The event yields and acceptances are affected by the choice of the parton distribution functions (PDFs) and the QCD factorization and renormalization scale uncertainties. The effects of the PDF choice on the acceptance and normalization of the ${\mathrm{{{\mathrm{Z}}_{\mathrm{}}^{\mathrm{}}}}}_{\mathrm{}}^{\mathrm{\prime }}$ signal are derived according to the PDF4LHC recommendations [67] and amount to 0.5% in the acceptance and 8–30% in the normalization of the signal, 0.2% in the acceptance and 4.7% in the normalization of the ${{\mathrm{V}}_{\mathrm{}}^{\mathrm{}}} {{\mathrm{V}}_{\mathrm{}}^{\mathrm{}}}$,${{\mathrm{V}}_{\mathrm{}}^{\mathrm{}}} {{\mathrm{H}}_{\mathrm{}}^{\mathrm{}}}$ background, and 0.1% in the acceptance and 0.1% in the normalization of the ${\mathrm{t}}_{\mathrm{}}^{\mathrm{}}$ $\overline{{{{\mathrm{t}}_{\mathrm{}}^{\mathrm{}}}}}$ background. The factorization and renormalization scale uncertainties are 3–15%, depending on the resonance mass for the signal, 18.9% for the ${{\mathrm{V}}_{\mathrm{}}^{\mathrm{}}} {{\mathrm{V}}_{\mathrm{}}^{\mathrm{}}}$,${{\mathrm{V}}_{\mathrm{}}^{\mathrm{}}} {{\mathrm{H}}_{\mathrm{}}^{\mathrm{}}}$ background, and 1% for the extrapolation of the top quark SFs to the SR.

The darkening of ECAL crystals, due to radiation damage, leads to a gradual timing shift, which was not properly propagated to the level 1 trigger for 2016 and 2017 [68]. This effect is accounted for by adding a 1% systematic uncertainty in the signal normalization. Additional systematic uncertainties come from estimations of the pileup contribution and the integrated luminosity [69–71]. A list of all systematic uncertainties is given in Table 4.

**Table 4:** Summary of systematic uncertainties for the background and signal samples. The entries labeled with $\dagger$ are also propagated to the shapes of the distributions. Uncertainties marked with $\ddagger$ impact the signal cross section. Uncertainties in the same line are treated as correlated. All uncertainties except for in the integrated luminosity are considered correlated across the three years of data taking

	${{\mathrm{V}}_{\mathrm{}}^{\mathrm{}}} \text {+jets}$	${\mathrm{t}}_{\mathrm{}}^{\mathrm{}}$ $\overline{{{{\mathrm{t}}_{\mathrm{}}^{\mathrm{}}}}}$, single top quark	${{\mathrm{V}}_{\mathrm{}}^{\mathrm{}}} {{\mathrm{V}}_{\mathrm{}}^{\mathrm{}}}$, ${{\mathrm{V}}_{\mathrm{}}^{\mathrm{}}} {{\mathrm{H}}_{\mathrm{}}^{\mathrm{}}}$	Signal
Bkg. normalization	6–40%	–	–	–
Top quark background SFs	–	0.4–9.5%	–	–
Electron id., isolation	–	–	3.6%	
Muon id., isolation	–	–	4.9%	
Electron trigger	–	–	0.9%	
Muon trigger	–	–	7%	
Lepton scale and resolution $\dagger$	–	–	–	1–3%
$p_{\mathrm {T}} ^\text {miss}$ scale and resolution	–	–	1%	
Jet energy scale $\dagger$	–	–	1.0%	1.0%
Jet energy resolution $\dagger$	–	–	0.1%	0.1%
Jet mass scale	–	–	0.6%	0.6%
Jet mass resolution	–	–	9.0%	9.0%
Higgs boson tagging	–	–	–	6%
${\mathrm{b}}_{\mathrm{}}^{\mathrm{}}$ tagging	–	1.4% ($0\ell$)	0.6% ($\le$1${\mathrm{b}}_{\mathrm{}}^{\mathrm{}}$), 6.5% (2${\mathrm{b}}_{\mathrm{}}^{\mathrm{}}$)	1-6% ($\le$1${\mathrm{b}}_{\mathrm{}}^{\mathrm{}}$), 4-15% (2${\mathrm{b}}_{\mathrm{}}^{\mathrm{}}$)
PDF, normalization	–	0.1%	4.7%	8–30% $\ddagger$
PDF, acceptance	–	0.1%	0.2%	0.5%
QCD renormalization and factorization scales	–	–	18.9%	3–15% $\ddagger$
Factorization and renorm. scales extrapolation	–	1%	–	–
Level 1 trigger	–	–	–	1%
Pileup	–	–	0.1%	0.1%
Integrated luminosity	–	–	1.8%	1.8%

## Results

Results are obtained from a combined profile likelihood fit to the unbinned $m_{{{\mathrm{X}}_{\mathrm{}}^{\mathrm{}}}}^{\text {T}}$ and $m_{{{\mathrm{X}}_{\mathrm{}}^{\mathrm{}}}}$ distributions of signal and background, shown in Figs. 5 and 6. Systematic uncertainties are treated as nuisance parameters and are profiled in the statistical interpretation [72–74]. The uncertainties in the signal normalization that are derived from the signal cross section are not profiled in the likelihood, and are reported separately as the uncertainty band of the theoretical cross section. The statistical methods, including the treatment of the nuisance parameters, are described in more detail in Ref. [16].

The background-only hypothesis is tested against a hypothesis also considering ${{\mathrm{{{\mathrm{Z}}_{\mathrm{}}^{\mathrm{}}}}}_{\mathrm{}}^{\mathrm{\prime }}} \rightarrow {{\mathrm{Z}}_{\mathrm{}}^{\mathrm{}}} {{\mathrm{H}}_{\mathrm{}}^{\mathrm{}}}$ signal in all categories. A modified frequentist method is used to determine 95% confidence level (CL) upper limits on the product of cross section and branching fraction as a function of $m_{{{\mathrm{X}}_{\mathrm{}}^{\mathrm{}}}}$, in which the distribution of the profile likelihood test statistic is derived using an asymptotic approximation [75].

**Fig. 7:**
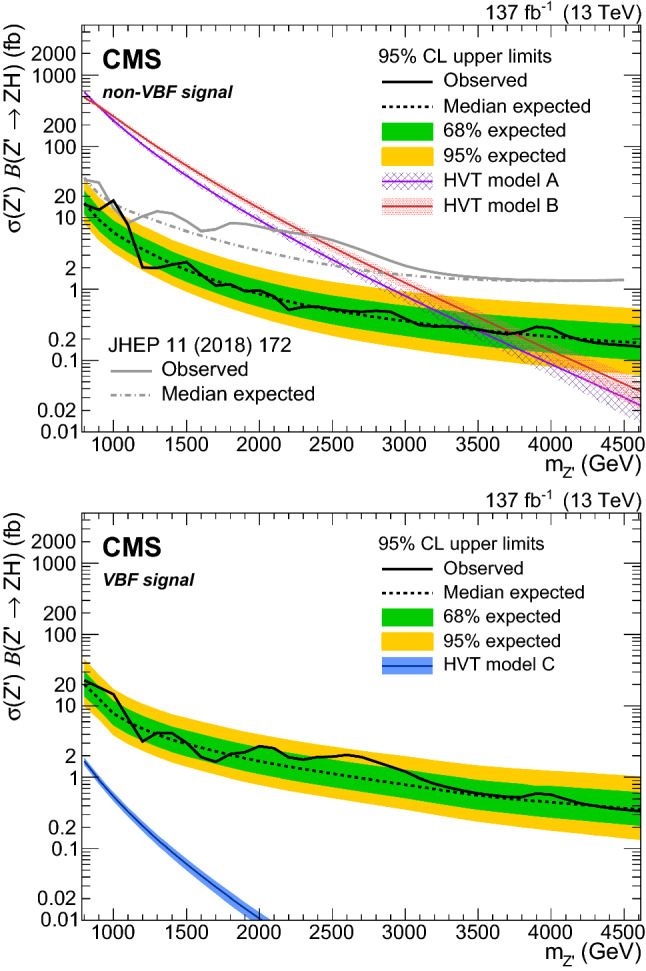
Observed and expected 95% CL upper limit on $\sigma \mathcal {B}({{\mathrm{{{\mathrm{Z}}_{\mathrm{}}^{\mathrm{}}}}}_{\mathrm{}}^{\mathrm{\prime }}} \rightarrow {{\mathrm{Z}}_{\mathrm{}}^{\mathrm{}}} {{\mathrm{H}}_{\mathrm{}}^{\mathrm{}}} )$ with all categories combined, for the non-VBF signal (upper) and VBF signal (lower), including all statistical and systematic uncertainties. The inner green band and the outer yellow band indicate the regions containing 68 and 95%, respectively, of the distribution of expected limits under the background-only hypothesis. The solid curves and their shaded areas correspond to the product of the cross section and the branching fractions predicted by the HVT models A and B (upper) and HVT model C (lower), and their relative uncertainties. The CMS search for a heavy resonance using 2016 data and the same final state [14] is shown as a comparison

The exclusion limits on the product of resonance cross section and branching fraction $\mathcal {B}({{\mathrm{{{\mathrm{Z}}_{\mathrm{}}^{\mathrm{}}}}}_{\mathrm{}}^{\mathrm{\prime }}} \rightarrow {{\mathrm{Z}}_{\mathrm{}}^{\mathrm{}}} {{\mathrm{H}}_{\mathrm{}}^{\mathrm{}}} )$ are reported as a function of the resonance mass in Fig. 7 for all categories, separately for the non-VBF and the VBF signals. The $2\ell$ categories dominate the sensitivity for heavy resonance masses smaller than 1$\,\text {Te}\text {V}$ because of the smaller backgrounds combined with the better experimental resolution; at larger masses, the $0\ell$ categories are more sensitive thanks to the larger branching fraction of the ${\mathrm{Z}}_{\mathrm{}}^{\mathrm{}}$ boson to neutrinos. The exclusion limits are shown up to 4.6$\,\text {Te}\text {V}$, which corresponds to the event with the highest $m_{{{\mathrm{X}}_{\mathrm{}}^{\mathrm{}}}}$ or $m_{{{\mathrm{X}}_{\mathrm{}}^{\mathrm{}}}}^{\text {T}}$ observed either in the SB or SR.

The largest excess for the non-VBF signal, corresponding to a local significance of 3 standard deviations, is observed at $m_{{{\mathrm{X}}_{\mathrm{}}^{\mathrm{}}}} =1\,\text {Te}\text {V}$. A ${\mathrm{{{\mathrm{Z}}_{\mathrm{}}^{\mathrm{}}}}}_{\mathrm{}}^{\mathrm{\prime }}$ boson with a mass smaller than 3.5$\,\text {Te}\text {V}$ is excluded at 95% CL in HVT model A, and a ${\mathrm{{{\mathrm{Z}}_{\mathrm{}}^{\mathrm{}}}}}_{\mathrm{}}^{\mathrm{\prime }}$ with mass smaller than 3.7$\,\text {Te}\text {V}$ is excluded in model B. The upper limit of the excluded mass range is increased by 0.85 (0.87)$\,\text {Te}\text {V}$ and 1.3 (1.4)$\,\text {Te}\text {V}$) in HVT model A (model B) compared to searches using 2016 data and the same final state by the ATLAS and CMS Collaborations, respectively [14, 15]. If the ${\mathrm{{{\mathrm{Z}}_{\mathrm{}}^{\mathrm{}}}}}_{\mathrm{}}^{\mathrm{\prime }}$ couples only to the SM bosons and is produced exclusively through VBF as in HVT model C, the data set analyzed is not large enough to exclude any range of mass. Upper limits on the product of the cross section and branching fraction are set between 23 and 0.3$\,\text {fb}$ for a ${\mathrm{{{\mathrm{Z}}_{\mathrm{}}^{\mathrm{}}}}}_{\mathrm{}}^{\mathrm{\prime }}$ mass between 0.8 and 4.6$\,\text {Te}\text {V}$, respectively.

**Fig. 8:**
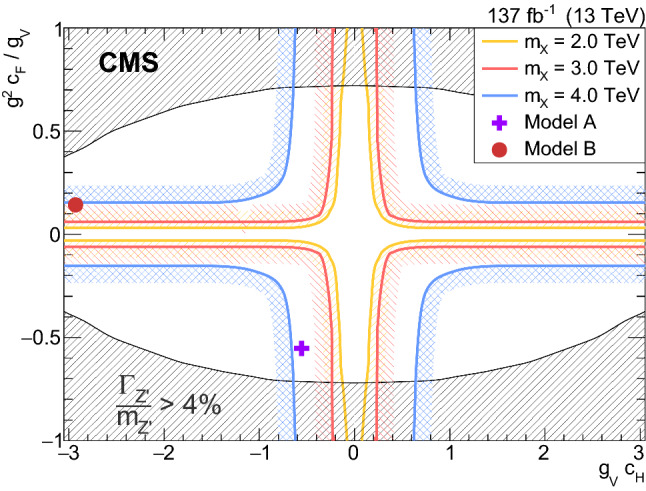
Observed exclusion limit in the space of the HVT model parameters [$g_\text {V} c_\text {H}$, $g^2c_\text {F}/g_\text {V}$], described in the text, for three different mass hypotheses of 2.0, 3.0, and 4.0$\,\text {Te}\text {V}$ for the non-VBF signal. The shaded bands indicate the side of each contour that is excluded. The benchmark scenarios corresponding to HVT models A and B are represented by a purple cross and a red point, respectively. The region of the parameter space where the natural resonance width ($\varGamma _{Z'}$) is larger than the typical experimental resolution of 4%, for which the narrow-width approximation is not valid, is shaded in grey

The exclusion limit of the non-VBF signal shown in Fig. 7 (upper) can be interpreted as a limit in the space of the HVT model parameters [$g_\text {V} c_\text {H}$, $g^2c_\text {F}/g_\text {V}$]. Combining all categories, the excluded region in such a parameter space for narrow resonances is shown in Fig. 8. The region of parameter space where the natural resonance width is larger than the typical experimental resolution of 4%, for which the narrow width assumption is not valid, is shaded.

## Summary

A search for a heavy resonance with a mass between 0.8 and 5.0$\,\text {Te}\text {V}$, decaying to a ${\mathrm{Z}}_{\mathrm{}}^{\mathrm{}}$ boson and a Higgs boson, has been described. The data samples were collected by the CMS experiment in the period 2016–2018 at $\sqrt{s}=13\,\text {Te}\text {V}$ and correspond to an integrated luminosity of 137$\,\text {fb}^{-1}$. In the final states explored the ${\mathrm{Z}}_{\mathrm{}}^{\mathrm{}}$ boson decays leptonically, resulting in events with either zero or two electrons or muons. Higgs bosons with a large Lorentz boost are reconstructed via their decays to hadrons. For models with a narrow spin-1 resonance, a new heavy vector boson ${\mathrm{{{\mathrm{Z}}_{\mathrm{}}^{\mathrm{}}}}}_{\mathrm{}}^{\mathrm{\prime }}$ with mass below 3.5 and 3.7$\,\text {Te}\text {V}$ is excluded at 95% confidence level in models where the heavy vector boson couples predominantly to fermions and bosons, respectively. These are the most stringent limits placed on the Heavy Vector Triplet ${\mathrm{{{\mathrm{Z}}_{\mathrm{}}^{\mathrm{}}}}}_{\mathrm{}}^{\mathrm{\prime }}$ model to date. If the heavy vector boson couples exclusively to standard model bosons, upper limits on the product of the cross section and branching fraction are set between 23 and 0.3$\,\text {fb}$ for a ${\mathrm{{{\mathrm{Z}}_{\mathrm{}}^{\mathrm{}}}}}_{\mathrm{}}^{\mathrm{\prime }}$ mass between 0.8 and 4.6$\,\text {Te}\text {V}$, respectively. This is the first limit set on a heavy vector boson coupling exclusively to standard model bosons in its production and decay.

## Data Availability

This manuscript has no associated data or the data will not be deposited. [Authors’ comment: Release and preservation of data used by the CMS Collaboration as the basis for publications is guided by the CMS policy as written in its document “CMS data preservation, re-use and open access policy” (https://cms-docdb.cern.ch/cgi-bin/PublicDocDB/RetrieveFile?docid=6032&filename=CMSDataPolicyV1.2.pdf&version=2).]

## References

[1] ATLAS Collaboration, Observation of a new particle in the search for the standard model Higgs boson with the ATLAS detector at the LHC. Phys. Lett. B **716**, 1 (2012). 10.1016/j.physletb.2012.08.020. arXiv:1207.7214

[2] CMS Collaboration, Observation of a new boson at a mass of 125 GeV with the CMS experiment at the LHC. Phys. Lett. B **716**, 30 (2012). 10.1016/j.physletb.2012.08.021. arXiv:1207.7235

[3] CMS Collaboration, Observation of a new boson with mass near 125 GeV in pp collisions at $$\sqrt{s} = 7$$ and 8 TeV. JHEP **06**, 081 (2013). 10.1007/JHEP06(2013)081. arXiv:1303.4571

[4] ATLAS and CMS Collaboration, Combined measurement of the Higgs boson mass in pp collisions at $$\sqrt{s}=7$$ and 8 TeV with the ATLAS and CMS experiments. Phys. Rev. Lett. **114**, 191803 (2015). 10.1103/PhysRevLett.114.191803. arXiv:1503.07589 10.1103/PhysRevLett.114.191803 26024162

[5] CMS Collaboration, A measurement of the Higgs boson mass in the diphoton decay channel. Phys. Lett. B **805**, 135425 (2020). 10.1016/j.physletb.2020.135425. arXiv:2002.06398

[6] ATLAS Collaboration, Evidence for the spin-0 nature of the Higgs boson using ATLAS data. Phys. Lett. B **726**, 120 (2013). 10.1016/j.physletb.2013.08.026. arXiv:1307.1432

[7] ATLAS and CMS Collaboration, Measurements of the Higgs boson production and decay rates and constraints on its couplings from a combined ATLAS and CMS analysis of the LHC pp collision data at $$\sqrt{s} = 7$$ and 8 TeV. JHEP **08**, 045 (2016). 10.1007/JHEP08(2016)045. arXiv:1606.02266

[8] Han T, Logan HE, McElrath B, Wang L-T (2003). Phenomenology of the little Higgs model. Phys. Rev. D.

[9] Schmaltz M, Tucker-Smith D (2005). Little Higgs theories. Ann. Rev. Nucl. Part. Sci..

[10] Perelstein M (2007). Little Higgs models and their phenomenology. Prog. Part. Nucl. Phys..

[11] Contino R, Pappadopulo D, Marzocca D, Rattazzi R (2011). On the effect of resonances in composite Higgs phenomenology. JHEP.

[12] Marzocca D, Serone M, Shu J (2012). General composite Higgs models. JHEP.

[13] Bellazzini B, Csaki C, Serra J (2014). Composite Higgses. Eur. Phys. J. C.

[14] CMS Collaboration, Search for heavy resonances decaying into a vector boson and a Higgs boson in final states with charged leptons, neutrinos, and b quarks at $$\sqrt{s} = 13~{\rm TeV}$$. JHEP **11**, 172 (2018). 10.1007/JHEP11(2018)172. arXiv:1807.02826

[15] ATLAS Collaboration, Search for heavy resonances decaying into a $$W$$ or $$Z$$ boson and a Higgs boson in final states with leptons and $$b$$-jets in 36 fb$$^{-1}$$ of $$\sqrt{s} = 13$$ TeV $$pp$$ collisions with the ATLAS detector. JHEP **03**, 174 (2018). 10.1007/JHEP03(2018)174. arXiv:1712.06518 [Erratum: 10.1007/JHEP11(2018)051]

[16] CMS Collaboration, Combination of CMS searches for heavy resonances decaying to pairs of bosons or leptons. Phys. Lett. B **798**, 134952 (2019). 10.1016/j.physletb.2019.134952. arXiv:1906.00057

[17] ATLAS Collaboration, Combination of searches for heavy resonances decaying into bosonic and leptonic final states using 36 fb$$^{-1}$$ of proton-proton collision data at $$\sqrt{s} = 13$$ TeV with the ATLAS detector. Phys. Rev. D **98**, 052008 (2018). 10.1103/PhysRevD.98.052008. arXiv:1808.02380

[18] Dorigo T (2018). Hadron collider searches for diboson resonances. Prog. Part. Nucl. Phys..

[19] Pappadopulo D, Thamm A, Torre R, Wulzer A (2014). Heavy vector triplets: bridging theory and data. JHEP.

[20] Barger VD, Keung W-Y, Ma E (1980). A gauge model with light W and Z bosons. Phys. Rev. D.

[21] CMS Collaboration, Search for heavy resonances decaying into two Higgs bosons or into a Higgs boson and a W or Z boson in proton–proton collisions at 13 TeV. JHEP **01**, 051 (2019). 10.1007/JHEP01(2019)051. arXiv:1808.01365

[22] ATLAS Collaboration, Search for heavy resonances decaying to a W or Z boson and a Higgs boson in the $$q\overline{q}(^{\prime })b\overline{b}$$ final state in pp collisions at $$\sqrt{s}=13$$ TeV with the ATLAS detector. Phys. Lett. B **774**, 494 (2017). 10.1016/j.physletb.2017.09.066. arXiv:1707.06958

[23] CMS Collaboration, Search for heavy resonances that decay into a vector boson and a Higgs boson in hadronic final states at $$\sqrt{s} = 13$$ TeV. Eur. Phys. J. C **77**, 636 (2017). 10.1140/epjc/s10052-017-5192-z. arXiv:1707.01303 10.1140/epjc/s10052-017-5192-z PMC6959389 32011608

[24] ATLAS Collaboration, G. Aad and others, Search for resonances decaying into a weak vector boson and a Higgs boson in the fully hadronic final state produced in proton–proton collisions at $$\sqrt{s}=13$$ TeV with the ATLAS detector. Phys. Rev. D **102**, 112008 (2020). https://doi.org/10.1103/PhysRevD.102.112008. arXiv:2007.05293

[25] CMS Collaboration, The CMS experiment at the CERN LHC. JINST **3**, S08004 (2008). 10.1088/1748-0221/3/08/S08004

[26] CMS Collaboration, The CMS trigger system. JINST **12**, P01020 (2017). 10.1088/1748-0221/12/01/P01020. arXiv:1609.02366

[27] Alwall J (2014). The automated computation of tree-level and next-to-leading order differential cross sections, and their matching to parton shower simulations. JHEP.

[28] Alwall J (2008). Comparative study of various algorithms for the merging of parton showers and matrix elements in hadronic collisions. Eur. Phys. J. C.

[29] Lindert JM (2017). Precise predictions for V+ jets dark matter backgrounds. Eur. Phys. J. C.

[30] Nason P (2004). A new method for combining NLO QCD with shower Monte Carlo algorithms. JHEP.

[31] Frixione S, Nason P, Oleari C (2007). Matching NLO QCD computations with parton shower simulations: the POWHEG method. JHEP.

[32] Alioli S, Nason P, Oleari C, Re E (2010). A general framework for implementing NLO calculations in shower Monte Carlo programs: the POWHEG BOX. JHEP.

[33] Frederix R, Re E, Torrielli P (2012). Single-top t-channel hadroproduction in the four-flavour scheme with POWHEG and aMC@NLO. JHEP.

[34] Re E (2011). Single-top Wt-channel production matched with parton showers using the POWHEG method. Eur. Phys. J. C.

[35] Campbell JM, Ellis RK, Nason P, Re E (2015). Top-pair production and decay at NLO matched with parton showers. JHEP.

[36] Czakon M, Mitov A (2014). Top++: a program for the calculation of the top-pair cross-section at hadron colliders. Comput. Phys. Commun..

[37] NNPDF Collaboration, Parton distributions for the LHC run II. JHEP **04**, 040 (2015). 10.1007/JHEP04(2015)040. arXiv:1410.8849

[38] NNPDF Collaboration, Parton distributions from high-precision collider data. Eur. Phys. J. C **77**, (2017) 10.1140/epjc/s10052-017-5199-5. arXiv:1706.00428 10.1140/epjc/s10052-017-5199-5 PMC6956957 31997920

[39] Sjöstrand T (2015). An introduction to PYTHIA 8.2. Comput. Phys. Commun..

[40] Skands P, Carrazza S, Rojo J (2013). Tuning PYTHIA 8.1: the Monash 2013 Tune. Eur. Phys. J. C.

[41] CMS Collaboration, Event generator tunes obtained from underlying event and multiparton scattering measurements. Eur. Phys. J. C **76**, 155 (2016). 10.1140/epjc/s10052-016-3988-x. arXiv:1512.00815 10.1140/epjc/s10052-016-3988-x PMC4946872 27471433

[42] CMS Collaboration, Extraction and validation of a new set of CMS PYTHIA8 tunes from underlying-event measurements. Eur. Phys. J. C **80**, 4 (2020). 10.1140/epjc/s10052-019-7499-4. arXiv:1903.12179 10.1140/epjc/s10052-019-7499-4 PMC6944267 31976986

[43] CMS Collaboration, Investigations of the impact of the parton shower tuning in PYTHIA8 in the modelling of $${\rm t}\overline{{\rm t}}$$ and 13 TeV. CMS Physics Analysis Summary CMS-PAS-TOP-16-021, CERN (2016)

[44] GEANT4 Collaboration, Geant4–a simulation toolkit. Nucl. Instrum. Methods A **506**, 250 (2003). 10.1016/S0168-9002(03)01368-8

[45] CMS Collaboration, Particle-flow reconstruction and global event description with the CMS detector. JINST **12**, P10003 (2017). 10.1088/1748-0221/12/10/p10003. arXiv:1706.04965

[46] Cacciari M, Salam GP, Soyez G (2008). The anti-$${k_{\rm T}}$$ jet clustering algorithm. JHEP.

[47] Cacciari M, Salam GP, Soyez G (2012). FastJet user manual. Eur. Phys. J. C.

[48] Cacciari M, Salam GP, Soyez G (2008). The catchment area of jets. JHEP.

[49] CMS Collaboration, Pileup mitigation at CMS in 13 TeV data. JINST **15**, P09018 (2020). 10.1088/1748-0221/15/09/p09018. arXiv:2003.00503

[50] Cacciari M, Salam GP (2008). Pileup subtraction using jet areas. Phys. Lett. B.

[51] Bertolini D, Harris P, Low M, Tran N (2014). Pileup per particle identification. JHEP.

[52] CMS Collaboration, Jet energy scale and resolution in the CMS experiment in pp collisions at 8 TeV. JINST **12**, P02014 (2017). 10.1088/1748-0221/12/02/P02014. arXiv:1607.03663

[53] CMS Collaboration, Performance of missing transverse momentum reconstruction in proton–proton collisions at $$\sqrt{s}=13$$ TeV using the CMS detector. JINST **14**, P07004 (2019). 10.1088/1748-0221/14/07/p07004. arXiv:1903.06078

[54] Dasgupta M, Fregoso A, Marzani S, Salam GP (2013). Towards an understanding of jet substructure. JHEP.

[55] Butterworth JM, Davison AR, Rubin M, Salam GP (2008). Jet substructure as a new Higgs search channel at the LHC. Phys. Rev. Lett..

[56] Larkoski AJ, Marzani S, Soyez G, Thaler J (2014). Soft drop. JHEP.

[57] CMS Collaboration, Identification techniques for highly boosted W bosons that decay into hadrons. JHEP **12**, 017 (2014). 10.1007/JHEP12(2014)017. arXiv:1410.4227

[58] CMS Collaboration, Identification of heavy-flavour jets with the CMS detector in pp collisions at 13 TeV. JINST **13**, P05011 (2018). 10.1088/1748-0221/13/05/p05011. arXiv:1712.07158

[59] CMS Collaboration, Performance of electron reconstruction and selection with the CMS detector in proton–proton collisions at $$\sqrt{s} = 8$$$$\,\text{TeV}$$. JINST **10**, P06005 (2015). 10.1088/1748-0221/10/06/P06005. arXiv:1502.02701

[60] CMS Collaboration, Performance of the CMS muon detector and muon reconstruction with proton–proton collisions at $$\sqrt{s} = 13$$ TeV. JINST **13**, P06015 (2018). 10.1088/1748-0221/13/06/P06015. arXiv:1804.04528

[61] CMS Collaboration, Performance of reconstruction and identification of $$\tau $$ leptons decaying to hadrons and $$\nu \tau $$ in pp collisions at $$\sqrt{s} = 13$$$$\,\text{ TeV }$$. JINST **13**, P10005 (2018). 10.1088/1748-0221/13/10/p10005. arXiv:1809.02816

[62] CMS Collaboration, Search for a heavy resonance decaying into a Z boson and a vector boson in the $$\nu \overline{\nu }{\rm q}\overline{\rm q}$$ final state. JHEP **07**, 075 (2018). 10.1007/JHEP07(2018)075. arXiv:1803.03838

[63] CMS Collaboration, Search for a heavy resonance decaying into a Z boson and a Z or W boson in $$2\ell $$2q final states at $$\sqrt{s}=13$$ TeV. JHEP **09**, 101 (2018). 10.1007/JHEP09(2018)101. arXiv:1803.10093

[64] R.A. Fisher, *Statistical Methods for Research Workers* (Oliver and Boyd, Edinburgh, 1925). ISBN:0-05-002170-2

[65] M.J. Oreglia, A study of the reactions $$\psi ^\prime \rightarrow \gamma \gamma \psi $$. PhD thesis, Stanford University (1980). SLAC Report SLAC-R-236

[66] Baehr M (2008). Herwig++ physics and manual. Eur. Phys. J. C.

[67] Butterworth J (2016). PDF4LHC recommendations for LHC Run II. J. Phys. G.

[68] CMS Collaboration, Performance of the CMS Level-1 trigger in proton–proton collisions at $$\sqrt{s} = 13$$ TeV. JINST **15**, P10017 (2020). 10.1088/1748-0221/15/10/p10017. arXiv:2006.10165

[69] CMS Collaboration, CMS luminosity measurement for the 2016 data-taking period. CMS Physics Analysis Summary CMS-PAS-LUM-17-001, CERN (2016)

[70] CMS Collaboration, CMS luminosity measurement for the 2017 data-taking period at $$\sqrt{s}=13$$ TeV. CMS Physics Analysis Summary CMS-PAS-LUM-17-004, CERN (2017)

[71] CMS Collaboration, CMS luminosity measurement for the 2018 data-taking period at $$\sqrt{s}=13$$ TeV. CMS Physics Analysis Summary CMS-PAS-LUM-18-002, CERN (2018)

[72] Junk T (1999). Confidence level computation for combining searches with small statistics. Nucl. Instrum. Methods A.

[73] Read AL (2002). Presentation of search results: the $${\text{ CL}_{\text{ s }}}$$ technique. J. Phys. G.

[74] CMS and ATLAS Collaborations, LHC Higgs Combination Group, Procedure for the LHC Higgs boson search combination in Summer 2011. Technical Report CMS-NOTE-2011-005. ATL-PHYS-PUB-2011-11, CERN (2011)

[75] G. Cowan, K. Cranmer, E. Gross, O. Vitells, Asymptotic formulae for likelihood-based tests of new physics. Eur. Phys. J. C **71**, 1554 (2011). 10.1140/epjc/s10052-011-1554-0. arXiv:1007.1727 [Erratum: 10.1140/epjc/s10052-013-2501-z]

